# Do Extended Reality Interventions Benefit Patients Undergoing Elective Cardiac Surgical and Interventional Procedures? A Systematic Review and Meta‐analysis

**DOI:** 10.1111/jocn.17578

**Published:** 2024-12-12

**Authors:** Emma Harris, Steven Fenton, John Stephenson, Fiona Ewart, Salime Goharinezhad, Hyunkook Lee, Felicity Astin

**Affiliations:** ^1^ School of Health, Wellbeing and Social Care, Faculty of Wellbeing, Education and Language Studies The Open University Milton Keynes UK; ^2^ School of Computing and Engineering University of Huddersfield Huddersfield UK; ^3^ School of Human and Health Sciences University of Huddersfield Huddersfield UK

**Keywords:** anxiety, extended reality, interventional cardiology, pain, patient experience, patient outcomes, systematic review, virtual reality

## Abstract

**Background:**

Extended reality (XR) interventions have the potential to benefit patients undergoing elective cardiac surgical and interventional procedures. However, there are no systematic reviews with meta‐analyses to guide clinical care.

**Aim:**

To critically evaluate the evidence on the effectiveness of XR interventions on patient anxiety and pain and other associated outcomes.

**Design:**

Systematic review and meta‐analysis following the PRISMA 2020 statement.

**Data Sources:**

A systematic search of five databases (CENTRAL, CINAHL, MEDLINE, PsycInfo, Scopus) from inception to July 2023.

**Methods:**

Screening and data extraction was conducted independently by multiple reviewers. Stata (Version 17) was used to conduct meta‐analyses for patient anxiety and pain. Secondary patient outcomes were summarised in a synthesis. The Cochrane Risk of Bias (Version 2) tool was applied to trials and the NHLBI Study Quality Assessment tools to all other study designs.

**Results:**

Of the 3372 records identified, 22 were included, 10 of which were eligible for inclusion in the meta‐analyses. Fifty‐seven percent of randomised trials were rated as high risk of bias. Virtual reality (VR) was the only XR technology evaluated. VR significantly reduced pre‐procedural anxiety (standardised mean difference: −1.29; 95% confidence interval − 1.96, −0.62, *p* < 0.001), and peri‐procedural anxiety (standardised mean difference: −0.50; 95% confidence interval − 0.83, −0.18, *p* < 0.003) but did not reduce pain levels, compared with usual care. VR increased pre‐procedural knowledge and postsurgical physical and pulmonary function. VR interventions may also improve emotional wellbeing, care delivery and physiological outcomes, but evidence was inconsistent.

**Conclusions:**

XR potentially benefits cardiac patients undergoing elective invasive procedures and surgery by reducing pre‐ and peri‐procedural anxiety and increasing procedural knowledge and physical function.

**Relevance to Clinical Practice:**

Cardiac nurses' role can be supported by VR interventions to improve the patient experience and several aspects of patient care.

**Patient or Public Contribution:**

Not applicable as this is a systematic review.

**Summary**

- What does this paper contribute to the wider global clinical community?
  ○ VR has significant potential as an intervention to benefit patients undergoing elective cardiac surgical and interventional procedures by reducing pre‐ and peri‐procedural anxiety and increasing procedural knowledge and postoperative physical and pulmonary function.
  ○ Cardiac nurses' role can be supported by VR interventions to enhance psychosocial support and education for patients undergoing elective cardiac surgical and interventional procedures
  ○ The characteristics of the VR interventions, such as the level of immersion and interactivity, proposed mechanism of action, intervention delivery, fidelity and reported side effects are underreported.

## 1 Introduction

The global burden of cardiovascular diseases means that an estimated one in every 13 people is living with the disease (British Heart Foundation 2024). An estimated 1.5 million cardiac surgeries (Vervoort et al. 2021) and 46 million interventional cardiology procedures (iData Research 2021) are performed globally each year. These include 1) surgical procedures such as Coronary Artery Bypass Graft Surgery (CABG) or Surgical Valve Replacement (SVR) and 2) interventional procedures in a catheter laboratory such as coronary angioplasty, transcatheter aortic valve implantation (TAVI), ablation, insertion of implantable cardioverter defibrillators (ICDs) or pacemakers. Cardiac nurses play a vital role in providing safe and effective care to patients before, during and after such procedures (White et al. 2018). As a major workforce within the multidisciplinary team, nurses help to optimise patients' care experiences and outcomes (Neubeck et al. 2023). Digital technologies have the potential to improve care quality and enhance person‐centred care (Kim et al. 2023; World Health Organisation 2021). The nursing workforce can lead the implementation of digital interventions in clinical practice, but there is a lack of evidence to guide decisions about the implementation of such technologies (Booth et al. 2021).

Extended reality technology (XR) is an emerging digital intervention that shows promise for improving patient care and outcomes such as anxiety and pain (Zhang, Lu, and Khanduja 2023). XR technologies stimulate multiple senses (visual, audial, haptic) to augment and/or replace the ‘real world’ with a simulated virtual environment and/or virtual objects (Brigham 2017). For example, virtual reality (VR) usually comprises a head‐mounted display and headphones to block the user's perception of the physical environment and replace it with a three‐dimensional computer‐generated environment. Augmented reality enables the user to interact with virtual objects that are overlaid onto the ‘real world’ physical environment through a display device. Mixed reality combines elements of both technologies. Key elements of XR technologies are the degree of immersion and interactivity (Zhang and Song 2022). Immersion is the physical and mental perception of being present in a virtual environment, and interactivity is where the virtual environment responds to the physical actions of the user (e.g., the virtual environment moves with the user as they turn their head (Fealy et al. 2019). The more interactive the XR technology, the greater immersion into the virtual environment and the more distracted the user becomes from the ‘real world’ (Fairclough et al. 2020). Within the envelope of XR technologies, VR is the most immersive and the most common XR technology used in healthcare settings and has helped to facilitate patient education, relaxation, distraction, or physical and psychological rehabilitation (Bouraghi et al. 2023; Boyce et al. 2023; Gao, Wang, and Liu 2023).

Several systematic reviews and meta‐analyses have evaluated the use of VR in cardiology settings but with limited focus on its use before and during cardiac surgical and interventional procedures (Bouraghi et al. 2023; Chen et al. 2022; Kavradim, Yangöz, and Özer 2023). Seventy percent of studies in the reviews evaluated VR‐augmented cardiac rehabilitation and found that VR interventions reduced negative emotions (patient anxiety, depression, stress) and increased exercise capacity, and physical functioning greater than usual care, although the certainty of evidence was low (Bouraghi et al. 2023; Chen et al. 2022; Kavradim, Yangöz, and Özer 2023). Only four of the 50 studies included in the reviews evaluated the use of VR interventions before or during cardiac procedures. Three studies found reductions in pre‐procedural anxiety before coronary angiography, cardiac catheterisation and catheter ablation (Chang et al. 2021; Keshvari et al. 2021; Morgan et al. 2021). Reductions in heart rate and systolic pressure (Keshvari et al. 2021) and increased understanding of cardiac catheterisation were also reported (Morgan et al. 2021). In the remaining study, VR was compared with Kalinox (mixture of oxygen and nitrous oxide) during chest drain removal after cardiac surgery, with both groups receiving usual care analgesia (Laghlam et al. 2021). Anxiety before, during and after the procedure was not different between groups, and pain levels were worse in the VR group immediately post‐procedure. Inconsistent results were also reported in a narrative review that described the pre‐, peri‐ and post‐procedural application of VR on anxiety and pain levels in cardiac surgical and interventional procedures (Mathari et al. 2024). However, the lack of a systematic literature review and meta‐analysis limits the extent to which findings can guide clinical practice. Furthermore, the narrative review did not evaluate all types of XR technologies and whether interventions had beneficial effects on other patient outcomes.

To address this gap, we have conducted the first comprehensive systematic review of international research evidence to evaluate how XR interventions benefit patients undergoing common elective cardiac surgical and interventional procedures. This includes meta‐analyses evaluating the effectiveness of XR interventions on patient anxiety and pain, and a synthesis of other patient outcomes examined in the literature. Findings will potentially inform cardiology practice, education and policy.

## 2 Methods

### 2.1 Design

To support the quality and transparency of this review, the 2020 Preferred Reporting Items for Systematic Reviews and Meta‐Analyses (PRISMA) guidelines (Page et al. 2021) were followed (checklist in Data S2) and the protocol was registered a priori on PROSPERO (CRD42022306623).

### 2.2 Search Strategy

A systematic and comprehensive search of five academic databases (CENTRAL, CINAHL 1937‐, MEDLINE 1946‐, APA PsycInfo 1887‐, Scopus 1966‐) was conducted in July 2023. Search terms were developed in partnership with a subject‐specific librarian and piloted on MEDLINE (see Data S2 for full search terms). No date or language restrictions were applied.

### 2.3 Eligibility Criteria

Table 1. shows the eligibility criteria for inclusion of studies. Criterion were developed using the PICOS framework, which includes population, intervention, comparison, outcomes and study type. Studies that evaluated patient anxiety or pain were eligible for inclusion in the meta‐analyses. Studies that evaluated other patient outcomes were eligible for inclusion in the synthesis. Since there is no consensus on which patient outcomes to use to evaluate the benefits of XR interventions, a scoping search of studies evaluating XR interventions in cardiac procedures was conducted in MEDLINE (available on request from authors). Outcome measures from studies identified in the scoping search were extracted and mapped against the domains in the Core Outcome Measures in Effectiveness Trials (COMET) taxonomy, (Dodd et al. 2018) which is endorsed by Cochrane (Higgins et al. 2022). Studies were therefore eligible for inclusion in the synthesis if they evaluated outcomes matching COMET domains (see Table 1).

**TABLE 1:** Eligibility criteria for the inclusion of studies.

PICOS framework	Inclusion criteria	Exclusion criteria
Population	Patients (any age) scheduled, undergoing, or treated with one of the following common cardiac surgical or interventional procedures: ablation, cardiac resynchronisation therapy, cardioversion, coronary sinus reducer implantation, implantable cardiac defibrillator (ICD), implantable loop recorder or pacemaker implantation/insertion, percutaneous coronary intervention (PCI)/ coronary angiography/angioplasty, mitral or aortic valve repair or replacement, including valve annuloplasty and valvuloplasty, septal defect closure, transcatheter aortic valve implantation (TAVI), surgical valve replacement (SVR) and coronary artery bypass surgery (CABG)	Any other procedure not listed Cardiopulmonary resuscitation (CPR)
Intervention	Extended reality (XR) intervention delivered before (defined as the time when participants were on the waiting list for the procedure or immediately pre‐procedure), during or after (defined as the recovery period in hospital when participant was inpatients after procedures) cardiac surgery or an interventional procedure. Interventions delivered individually or in a group setting were eligible for inclusion. All types of XR intervention delivery methods were eligible for inclusion (e.g., head‐mounted display systems, glasses and headphones)	Music only where not incorporated within an XR intervention Interventions intended for health professionals or students Interventions intended for health professionals or students Interventions for clinical simulation training XR interventions delivered as part of outpatient cardiac rehabilitation
Control	Any	n/a
Outcomes	*Meta‐analyses outcomes: Studies evaluating patient anxiety or pain, pre‐, peri‐ or post‐cardiac surgical or interventional procedure, using any patient questionnaire (self‐reported or verbally administered by researcher or health professional)*	Qualitative data Outcomes related to the health professional
	*Patient outcomes eligible for inclusion in the synthesis (grouped by COMET domain):*	
	Emotional functioning/wellbeing (e.g., impact on emotions or overall wellbeing)	
	Cognitive functioning (e.g., patient knowledge‐related outcomes)	
	Physical functioning (e.g., impact on physical activities of daily living such as walking)	
	Delivery of care (e.g., adherence/compliance, patient preference, tolerability of intervention and satisfaction)	
	Physiological (e.g., haemodynamics and pulmonary function)	
Study type	Controlled trials, quasi‐experimental, pre‐post/post‐ intervention and observational studies (cross‐sectional studies, case–control studies, case series and case reports)	Literature reviews, trial protocols, conference presentations/proceedings, abstract only, editorials, letters, qualitative studies

### 2.4 Study Selection

Search results were managed with Covidence systematic review software (Veritas Health Innovation, Melbourne, Australia). Studies were screened for eligibility by title and abstract, before full‐text screening. The eligibility of each study was determined independently by two reviewers (EH and either FA, SF or HL) at each screening stage and any disagreements resolved by discussion.

### 2.5 Data Extraction

Data were independently extracted from all included studies by two reviewers (EH and either FA, FE or SF) using a standardised form on Microsoft Excel (version 16): first author, publication year, country, study design, sample size, participant characteristics, cardiac procedure, XR intervention details (type, timing, setting, content, duration, delivery), comparison groups, relevant patient outcomes (see Table 1) and results. Disagreements were resolved through discussions. For studies included in the meta‐analyses, primary outcome data were extracted by a statistician (JS) and checked by EH.

### 2.6 Quality Appraisal

The quality of all studies was independently assessed by two pairs of reviewers (EH and either SF or SG). Disagreements were resolved by discussion with another reviewer (FA). The Cochrane Risk of Bias 2 (RoB2) tool was used to assess bias in randomised controlled trials (RCTs) and randomised comparative trials across five domains (randomisation process, deviations from the intended interventions, missing outcome data, measurement of the outcome and selection of the reported result) (Sterne et al. 2019). Overall risk of bias for each study was rated as either ‘low’, ‘some concerns’ or ‘high’, according to the guidance. The protocol/analysis plan was requested from authors where required. To appraise non‐randomised studies, a modified version of the NHLBI Study Quality Assessment tool (NHLBI Risk Assessment Work Group 2013) was developed (Table S2). Rating scores between 0–3.5 points indicated poor quality, 4–6 points indicated some concerns, and 6.5–8 points indicated good quality.

### 2.7 Data Analysis and Synthesis

Data extracted from all studies were summarised in a table. All studies were assessed for inclusion in the meta‐analyses for the outcomes of patient anxiety and pain. Exclusion criteria for the meta‐analyses were absence of a usual care control group and outcome measures other than pain or anxiety. All other eligible outcomes (as defined in Table 1) were synthesised and presented in a narrative.

Following review of studies, three meta‐analyses were proposed to evaluate the effect of XR interventions on pre‐procedural anxiety, peri‐procedural anxiety and peri/post‐procedural pain. To decide which studies were eligible for each synthesis, the outcome measures and timing of the XR intervention (i.e., pre‐, peri‐, post‐cardiac procedure) for each study were summarised and discussed by two reviewers. All meta‐analyses were based on controlled trials comparing XR interventions with standard care. Standardised estimates were based on scores reported in XR groups minus scores in standard care groups. Intervention groups were combined for analysis in studies including multiple XR groups.

In studies that measured anxiety using both the State–Trait Anxiety Inventory (STAI) and Visual Analogue Scale (VAS), results from the STAI were preferentially included in the derivation of the synthesised estimate. In studies that reported the STAI‐State and STAI‐Trait scores (but did not provide the overall STAI score), the STAI‐State score was included in the derivation of the synthesised estimate.

Standard deviation (SD) values were used directly if quoted in included studies; and calculated from reported standard errors and/or confidence intervals (CIs) in studies that provided this information but did not provide SD values. In studies that provided the interquartile range (IQR) but not SD as a measure of data variability, SD was estimated from given IQR values. In studies that reported results graphically, means and SDs were determined from graphs.

Random effects meta‐analyses were constructed for each outcome using restricted maximum likelihood estimation methods. Random effects models were chosen to reflect recognised clinical and methodological heterogeneity across included studies with respect to all outcomes.

Forest plots were conducted for all meta‐analyses, reporting synthesised estimates and associated 95% CIs, and a Z‐test for the estimated effect. Heterogeneity statistics were also reported, including results from Cochran's Q test for heterogeneity, the *I* ^2^ statistic (proportion of variation across studies ascribed to heterogeneity) and the *τ* ^2^ statistic (an estimate of between‐study variance in random effects models).

Sensitivity analyses were proposed on meta‐analyses with three or more included studies to assess the robustness of the derived estimates. Any study which was suspected of excessive influence on the resulting influence plot (considered to be indicated by the point estimate of the ‘omitted’ analysis of a study lying outside the confidence interval of the ‘combined’ analysis) was flagged as an influential study. Funnel plots were proposed for meta‐analyses with 10 or more included studies to detect small study effect‐related bias; including publication bias and other types of bias which may result from the true treatment effect differing between small and large studies; however, funnel plots were not constructed due to an insufficient number of studies included in any of the meta‐analyses. All meta‐analyses were conducted using Stata Version 17 (StataCorp. 2021).

## 3 Results

### 3.1 Study Selection

**FIGURE 1:**
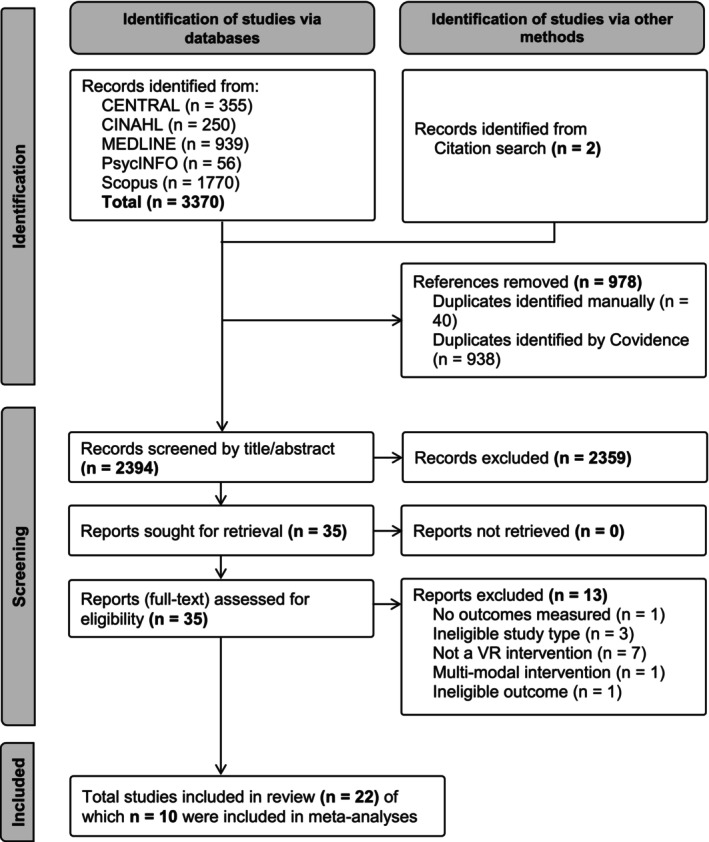
PRISMA flow‐diagram of search results and study selection.

The search results and study selection process are presented in Figure 1. In summary, the search yielded 3372 records. After duplicate removal, 2359 records were excluded via title and abstract screening, and 35 full‐text studies were retrieved. Following eligibility assessment, 13 studies were excluded, resulting in 22 studies included in the review, 10 of which were suitable for inclusion in the meta‐analyses.

### 3.2 Overview of Study Characteristics

Table 2 presents the characteristics and main results of the included studies. A range of different designs were identified; 12 RCTs, 2 randomised comparative trials, 2 non‐randomised controlled studies, 4 pre‐post‐test studies, 1 post‐test only study and 1 case study. Most studies (86%) were published after 2020 and were conducted across four continents; Europe (Aardoom et al. 2022; Bruno et al. 2020; Coulibaly et al. 2022; Grab et al. 2023; Laghlam et al. 2021; Lanquetuit et al. 2022; Lind et al. 2023; Morgan et al. 2021; Oudkerk Pool et al. 2022; Rousseaux et al. 2022; Roxburgh et al. 2021), South America (Cacau et al. 2013; Lima et al. 2020; Ribeiro et al. 2021), Asia (Chang et al. 2020, 2021; Keshvari et al. 2021; Pouryousef et al. 2020) and North America (Hendricks et al. 2020; Mosso‐Vázquez et al. 2014, 2013; Zablah et al. 2022). The sample size of RCTs ranged from 20 (Hendricks et al. 2020) to 180 (Laghlam et al. 2021), and from three (Zablah et al. 2022) to 99 (Roxburgh et al. 2021) for non‐randomised studies. Fourteen studies involved participants undergoing interventional cardiac procedures (Aardoom et al. 2022; Bruno et al. 2020; Chang et al. 2020, 2021; Coulibaly et al. 2022; Keshvari et al. 2021; Laghlam et al. 2021; Lanquetuit et al. 2022; Lind et al. 2023; Morgan et al. 2021; Oudkerk Pool et al. 2022; Pouryousef et al. 2020; Rousseaux et al. 2022; Zablah et al. 2022), six involved surgical cardiac procedures (Cacau et al. 2013; Grab et al. 2023; Hendricks et al. 2020; Lima et al. 2020; Ribeiro et al. 2021; Rousseaux et al. 2022), and two involved a mix of surgical and interventional cardiac procedures (Mosso‐Vázquez et al. 2014, 2013).

**TABLE 2:** Overview of included studies.

Clinical phase	Study (^a^indicates inclusion in meta‐analyses)	Study design	Participants	Cardiac procedure(s)	VR type; timing; delivery; duration; side effects/tolerance	Comparison groups	Main outcome measures	Main results
**Studies evaluating *VR‐facilitated education* interventions**								
Pre‐procedure	Aardoom et al. (2022), Netherlands	Post‐test only	Total *n* = 8 Age: 67 years (SD 7.5 years) 25% F	Interventional: Cardiac catheterisation	VR‐facilitated education Delivered 1–2 weeks pre‐procedure (home or clinic) VR head‐mounted display audio visual (Oculus Rift Go) 20 min approx duration Side effects: none	None	Satisfaction via CSQ‐8 (max score of 32. Higher scores indicate higher satisfaction) Feeling informed, better prepared for procedure, and reduction of negative psychological consequences (researcher generated)	Average satisfaction: 27.1 (SD 3.2) 100% agreed to feeling better informed 88% better prepared 88% felt negative psychological consequences reduced
Pre‐procedure	Chang et al. (2020), Taiwan	Pre‐test‐ post‐test	Total *n* = 32 Age 41 years (range 35–54 years); 44% F	Interventional: Catheter ablation for atrial fibrillation (AF)	VR‐facilitated education Delivered pre‐procedure within hospital (timing not reported) VR head‐mounted display audio visual 320 s duration Side effects: none	None	Researcher‐generated: Self‐efficacy statements for procedure‐related knowledge and anxiety (pre‐procedural) Satisfaction with educational materials Knowledge about procedure and complications (4‐item)	Less anxious after VR (Pre‐VR 27%; Post‐VR 80% reported not feeling anxious) Improved familiarity with procedure after VR (Pre‐VR 23%; Post‐VR 88%) and greater confidence in knowledge of procedure (Pre‐VR 34%; Post‐VR 86%) 92% willing to have VR again; 89% would recommend VR to others Greater knowledge after VR (Percentage who had full knowledge scores: Pre‐VR 35%; Post‐VR 78%). No p‐values reported
Pre‐procedure	Chang et al. (2021)^a^, Taiwan	RCT	Total *n* = 33 **VR** *n* = 11; Age > 40 years; 82% F **Control** *n* = 22; Age > 40 years; 36% F	Interventional: Catheter ablation for AF	VR‐facilitated education and discussion with HCP Delivered pre‐procedure within hospital (timing not reported) VR head‐mounted display audio visual 3 min duration Side effects: Not reported	**Control:** Usual care (paper‐based educational material same content as VR and discussion with HCP)	Researcher‐generated: Self‐efficacy statements (pre‐procedural anxiety, knowledge) on 10‐point Likert scale Satisfaction (educational materials met your needs) on 10‐point Likert scale Knowledge about procedure and complications (4‐item)	Less anxious post‐VR (feeling not anxious: 9.1/10) than control (feeling not anxious: 6.4) *p* < 0.05^b^ ^,^ ^c^ Higher satisfaction with education post‐VR (8.8/10) than control (6.7/10) *p* < 0.05^c^ Greater knowledge post‐VR than control for all 4 questions *p* < 0.05^c^ Improved familiarity with procedure post‐VR (8.9/10) than control (7.0/10) *p* < 0.05^c^ Greater confidence in procedure post‐VR (8.4/10) than control (6.8/10) *p* < 0.05^c^
Pre‐procedure	Grab et al. (2023)^a^, Germany	RCT	Total *n* = 99 **VR** *n* = 31; Age: 66 years (SD 8 years); 16% F **3D‐model** *n* = 34; Age: 66 years (SD 10 years); 15% F **Control** *n* = 34; Age 63 years (SD 14 years); 6% F	Surgical: Coronary artery bypass graft (CABG); Surgical aortic valve replacement (SAVR); Thoracic aortic aneurysm repair (TAA)	VR‐facilitated education Delivered pre‐procedure within hospital (timing not reported) VR goggles (Oculus Quest 2) Visual only with concurrent Dr. explanation 21.6 (SD 3.6) min duration Side effects: *n* = 2 (6%) experienced dizziness using VR and were excluded from analysis	**3D‐model:** Dr. explanation using physical models of heart anatomy to help explain the procedure. Duration: 20.3 (SD 3.2) min **Control:** Usual care. Dr. explanation using usual care paper information of heart anatomy. Duration: 19.6 (SD 3.8) min	Pre‐procedural anxiety via 10‐point Visual Analogue Scale (VAS) and State–Trait‐Anxiety Inventory (STAI) Knowledge of procedure (researcher‐generated) Satisfaction with and quality of patient education (PE) material and with physician delivering education	Lower pre‐procedural anxiety via STAI post‐VR (23) than control (25.6) *p* < 0.05^b^ Reduced pre‐procedural anxiety via VAS post‐VR (4.32) than pre‐VR (5), *p* < 0.0001^c^. NS in other groups Greater knowledge post‐education in all groups^c^ Greater procedural understanding post‐VR (92, SD 8%) than control (85, SD 8%). *p* = 0.011^c^ Higher procedural knowledge correlated with lower VAS pre‐procedural anxiety (B = −0.197, *p* = 0.001)^c^ Higher satisfaction with PE post‐VR (93, SD 10%) than control (85, SD 13%), *p* < 0.004^c^ Satisfaction with physician not different between groups
Pre‐procedure	Morgan et al. (2021)^a^, UK	RCT	Total *n* = 64 Age 68.7 years (range 38–84 years); 42% F **VR** *n* = 33 **Control** *n* = 31	Interventional: Cardiac catheterisation	VR‐facilitated education plus usual care written and verbal info Delivered 1‐week pre‐procedure within hospital VR head‐mounted display (concurrent audio through earplugs) 10 min duration Side effects: not reported	**Control:** Usual care (BHF info leaflets, verbal explanation of procedure by pre‐procedural assessment nurse) plus BHF video of procedure (unknown duration)	Pre‐procedural anxiety via 6‐item questionnaire based on STAI Perceived understanding of rationale for procedure and how procedure was done via 4‐point rating scales Rating for having enough information via 4‐point scale. Satisfaction with preparation and information prior to procedure (0–10 rating scale)	Lower pre‐procedural anxiety post‐VR than control but NS between groups, *p* > 0.05^b^ Perceived understanding of procedure rationale not different post‐VR than control Greater perceived understanding of how procedure was done post‐VR (3.88) than control (3.23) *p* < 0.01^c^ Received sufficient info not different post‐VR than control Satisfaction with preparation and info not different post‐VR than control
Pre‐procedure	Oudkerk Pool et al. (2022)^a^, The Netherlands	RCT	Total *n* = 50 **VR** *n* = 25; Age 44.5 years (SD 9.9 years); 56% F **Control** *n* = 25; Age 43.1 years (SD 12 years); 48% F	Interventional: Transcutaneous Atrial Septum Defect closure; Transcutaneous Patent Foramen Ovale closure	VR‐facilitated education plus usual care Delivered 1‐week pre‐procedure within hospital. Link to film used in VR could also be viewed at home VR head‐mounted display (Oculus Go, no audio details) 5 min duration Side effects: not reported	**Control:** usual care (explanation of procedure from cardiologist and informative flyer on procedure)	Pre‐procedural anxiety via STAI and Amsterdam Preoperative Anxiety and Information Scale (APAIS) administered post‐VR/control and 1‐week prior to procedure	Lower pre‐procedural anxiety via STAI‐state (1‐week pre) in VR group (38.8, SD 7.3) than control (45.3, SD 10.6) *p* < 0.05^b^ APAIS anxiety score (1‐week pre) not different between groups APAIS need for information score (1‐week pre) not different between groups
**Studies evaluating *VR‐based relaxation* interventions**								
Pre‐procedure	Keshvari et al. (2021)^a^, Iran	RCT	Total *n* = 40 **VR** *n* = 40; Age 50.95 years (SD 4.12 years); 20% F **Control** *n* = 40; Age 52 years (SD 4 years); 37.5% F	Interventional: Diagnostic coronary angiography	VR‐based relaxation Delivered immediately before procedure within hospital VR video headset (Remix company) and Huawei mobile phone with headphone (audio) 5 min duration Side effects: not reported	**Control:** Usual care (no VR)	Pre‐procedural anxiety via STAI Heart rate (HR), respiratory rate (RR), blood pressure (BP)	Lower pre‐procedural anxiety post‐VR (13.1, SD 2.1) than control (15.1, SD 3.5) *p* < 0.05^b^ ^,^ ^c^ Lower HR post‐VR (69, SD 4 bpm) than control (74, SD 4.2 bpm) *p* = 0.035)^c^ RR not different between groups Lower SBP post‐VR (125, SD 18 mmHg) than pre‐VR (136, SD 21 mmHg) *p* = 0.016^c^ but not different between groups
Pre‐procedure	Pouryousef et al. (2020)^a^, Iran	RCT	Total *n* = 90 **VR** *n* = 30; Age 50 years (SD 8.1 years) **Rhythmic breathing** *n* = 30; Age 50.6 years (SD 8.2 years) **Control** *n* = 30; Age 51.4 years (SD 8.1 years)	Interventional: Coronary angiography	VR‐based relaxation Delivered 1‐h before procedure within hospital VR (no details headset) 5 min duration Side effects: not reported	**Rhythmic breathing (RB)**: Sukha Pranayama technique (slow breathing through nostrils) 5 min duration **Control:** usual care (no VR or RB)	Pre‐procedural anxiety via STAI (pre, 30 min post, 1 h post‐VR, RB, or control)	Lower pre‐procedural anxiety at 30 min post‐VR (41.1, SD 7.2) than RB (47.6, SD 5.5) and control (55.8, SD 6.4) *p* = 0.001^c^ Lower pre‐procedural anxiety at 1 h post‐VR (42.3, SD 7.5) than RB (47.5, SD 6.1) and control (55.9, SD 6.5) *p* < 0.05^b^ ^,^ ^c^
Pre‐ and post‐ procedure	Rousseaux et al. (2022)^a^, Belgium	RCT	Total *n* = 100 **VR** *n* = 25; Age 64.7 years (SD 13.4 years); 24% F **Hypnosis** *n* = 25; Age 67.6 years (SD 12.5 years); 28% F **VR hypnosis** *n* = 25; Age 68.4 years (SD 7.8 years); 16% F **Control** *n* = 25; Age 63.3 years (SD 11.5 years); 28% F	Surgical: CAGB; mitral or aortic valve replacement; others	VR‐based relaxation plus usual care Delivered twice: 1‐day pre‐ (hospital ward) and 1‐day post‐ cardiac procedure (in ICU) VR head‐mounted display with goggles (Oncomfort) audio visual 20 min duration Side effects: not reported	**Control:** Usual care (no VR or hypnosis): paracetamol plus spinal morphine when required **Hypnosis:** usual care plus 20 min hypnosis audio track **VR hypnosis (VRH):** usual care plus VR with hypnosis audio integrated (20 min)	Pre‐procedural anxiety via 10‐point VAS Post‐procedural pain via 10‐point VAS Pre‐ and post‐procedural fatigue and relaxation HR, RR, and arterial pressure via bed monitor	Lower pre‐procedural anxiety post‐VR (2.52, SD 1.33) than control (4.54, SD 1.61) *p* < 0.05^b^ ^,^ ^c^ Higher post‐procedural pain with VR (4.19, SD 6.37) than control (3.2, SD 6.94) but NS, *p* > 0.05^b^. No difference in pain medication between groups *p* > 0.05. Fatigue not different between groups at any time. Relaxation not different between groups at any time. Higher HR post‐procedure than pre‐procedure in all groups *p* < 0.0001^c^ Lower arterial pressure at baseline than other timepoints in all groups *p* < 0.005^c^ RR not different between groups at any time
Peri‐procedure	Bruno et al. (2020)^a^, Germany	RCT	Total *n* = 32 **VR** *n* = 16; Median age 82 years (IQR 78.3–87 years); 31.2% F **Control** *n* = 16; Median age 83 years (IQR 78.3–86.8 years); 43.7% F	Interventional: Transcatheter aortic valve implantation (TAVI)	VR‐based relaxation plus usual care Delivered peri‐procedure VR head‐mounted display (Medion Erazer X1000 MR Glasses) no audio details Median VR duration 30.5 min (IQR 23.5–46) Tolerance: Headset removal due to annoyance and heat (not related to VR) 81% wore headset until valve implant, 37.5% wore until end of procedure; cybersickness: none	**Control:** Usual care (no VR): conscious sedation, analgesia given when required	Peri‐procedural anxiety measured 1‐day post‐procedure via 10‐point VAS Peri‐procedural pain measured post‐procedure via 10‐point VAS Satisfaction with VR Peri‐procedural BP fluctuations	Lower peri‐procedural anxiety in VR group (median 2, IQR 0–3.75) than control (median 5, IQR 2–8), *p* < 0.05^b^ ^,^ ^c^ Peri‐procedural pain not different between groups, p > 0.05^b^ ^,^ ^c^ (median: 4 in both groups). Analgesia and sedation dose and rates NS between groups *p* = 0.08. 93.8% would use VR again BP increases/ decreases not different between groups
Peri‐procedure	Coulibaly et al. (2022)^a^, France	Non‐randomised control trial	Total *n* = 86 **VR** *n* = 25; Age 61 years (SD 17 years); 24% F **Control** *n* = 61; Age 69 years (SD 14 years); 22% F	Interventional: EP or cardiac pacing procedure	VR‐based relaxation plus usual care Delivered peri‐procedure VR headset with audio headset (PICO Goblin2, Cayceo) Procedure duration 56, (SD 32) min Tolerance: 30% removed headset during procedure (most due to discomfort); cybersickness: none	**Control:** Usual care (no VR): sedation and analgesia given when required. Procedure duration 46, (SD 29) min	Post‐procedural pain and comfort measured immediately post‐procedure and at discharge via 10‐point VAS	Post‐procedural pain not different post‐VR (2.11, SD 2.9) than control (2.57, SD 2.88) p > 0.05^b^ Analgesia NS different between groups. Lower post‐procedural comfort in VR group (7.8, SD 1.8) than control (9.5, SD 1.2) *p* < 0.01^c^ Lower comfort at discharge in VR group (7.9, SD 2.1) than control (9.7, SD 0.7) *p* < 0.01^c^
Peri‐procedure	Laghlam et al. (2021), France	Randomised comparative trial	Total *n* = 180 **VR** *n* = 90; Median age 68 years (IQR 61.5–75 years); 31% F **Kalinox** *n* = 90; Median age 68 years (IQR 60–74 years); 20% F	Interventional: Withdrawal of mediastinal and pleural drains 2‐days after cardiac surgery	VR‐based relaxation plus usual care analgesics Delivered peri‐procedure. Started 5 min before until 10 min after procedure (97% on ICU, 3% on ward) VR headset (Deepsen, DT Didier) no audio details Unknown duration Tolerance: *n* = 2 removed VR and excluded Side effects: 3% *n* = 2 vertigo; *n* = 1 nausea	**Kalinox** (Air Liquide, Paris France) equimolar mixture of oxygen and nitrous oxide plus usual care analgesics. Delivered continuously from 1 min until 1 min after procedure Side effects: *n* = 2 euphoria, *n* = 1 headache	Post‐procedural anxiety immediately and 10 min post‐procedure via 0–10 numerical scale Post‐procedural pain immediately and 10 min post‐procedure via 0–10 Peri‐procedural pain via numerical scale analgesia/nociception index (ANI) monitor (higher ANI = lower pain) Satisfaction with procedure	Post‐procedural anxiety not different between groups Higher post‐procedural pain with VR (median 5, IQR 3–7) than Kalinox (median 3, IQR 2–6) *p* = 0.009^c^ immediately after but not different 10‐min post‐procedure. Peri‐procedural pain not different between groups Less satisfied with procedure in VR (10% not very satisfied) than Kalinox (1%) p = 0.01^c^
Peri‐procedure	Lanquetuit et al. (2022), France	Pre‐test‐ post‐test	Total *n* = 49 Age: 52.3 years (SD 18.4 years); 47% F	Interventional: ablation, pacemaker insertion, pericardial drainage, angiogram, EP studies, foramen ovale closure	VR‐based relaxation plus usual care analgesics when required Delivered peri‐procedure VR head‐mounted display audio visual (2 different headsets available: HypnoVR or DeepsenVR) 67% had HypnoVR device (duration 10, 20 min or indefinite); 33% had DeepsenVR device (duration 15, 30, or 60 min) Side effects: 12% removed headset during procedure due to cybersickness or discomfort	None	Peri‐ and post‐procedural anxiety measured post‐procedure via 0–10 scale Peri‐ and post‐procedural pain and discomfort measured post‐procedure via 0–10 scale Satisfaction with procedure and healthcare team via 0–10 scale	Anxiety reduced from pre (5.8, SD 3.4) peri (2.9, SD 2.6) to post‐procedure (0.7, SD 1.8) *p* < 0.0001^c^ Pain increased from pre (0.1, SD 0.4) peri (2.6, SD 2.5) to post‐procedure (1.2, SD 2.3) *p* < 0.05^c^ Analgesia used by 18.4% and sedation by 40.8% of participants. Discomfort was low and did not change post‐procedure Procedural satisfaction: 9 (SD 1.5). 96% would reuse VR Satisfaction with healthcare team: 9.1 (SD 1.9)
Post‐procedure	Mosso‐Vázquez et al. (2013), Mexico	Pre‐test post‐test	Total *n* = 22; Age 56.9 years (SD 10.3 years); 32% F	Interventional or surgical: SVR, coronary angioplasty/stenting, valvuloplasty or repair, ventricular septal defect repair	VR‐based relaxation Delivered within 24 h post‐procedure in ICU Head‐mounted display (eMagin Corporation) no audio details VR duration 30 min Tolerance: 3 (14%) patients removed VR due to cybersickness	None	Discomfort via 0–10 Likert scale HR, RR, BP	95% reported less discomfort post‐VR compared to pre‐VR (no p‐values) RR reduced in 64% of patients post‐VR HR: not sensitive to change BP: reduced diastolic pressure in 64% of patients post‐VR
Post‐procedure	Mosso‐Vázquez et al. (2014), Mexico	Pre‐test post‐test	Total *n* = 67; Age not reported; 37% F	Interventional or surgical: valve replacement or repair surgery, coronary angioplasty, valvuloplasty/repair, ventricular septal defect repair	Same as above	None	Pain via 0–10 Likert scale HR, RR, mean arterial pressure (MA)	Pain: 88% reported less pain. Mean change from pre‐ to post‐VR: 3.75 (no p‐values) HR reduced in 37% of patients post‐VR RR reduced in 64% of patients post‐VR MAP reduced in 52% of patients post‐VR
**Studies evaluating *VR‐based distraction* interventions**								
Pre‐procedure	Hendricks et al. (2020), USA	Pilot randomised comparative trial	Total *n* = 20 median age 70 years (range 53–77 years). **VR** *n* = 10; 10% F **Non‐VR** *n* = 10; 10% F	Surgical: CABG (first‐time sternotomy)	VR‐based distraction Delivered 20 min before procedure (within hospital) VR head‐mounted display with audio headset (Samsung Gear Occulus) 20 min duration Side effects: not reported	**Non‐VR:** tablet‐based game (CandyCrush)	Pre‐procedural anxiety via STAI and sub‐scores	Total ‘anxiety present’ score lower post‐VR (15, SD 1.3) than pre‐VR (19, SD 1.7). p = 0.03^c^. No difference in control group Feeling tense, strained, and calm improved post‐VR than non‐VR. *p* < 0.01^c^ Feeling steady worse post‐VR than non‐VR. *p* < 0.05^c^
Peri‐procedure	Lind et al. (2023)^a^, Germany	RCT	Total *n* = 117 **VR** *n* = 59; Age 81.1 years (SD 5.7 years); 49.2% F **Control** *n* = 58; Age 81.2 years (SD 5.5 years); 50% F	Interventional: TAVI	VR‐based distraction plus usual care Delivered peri‐procedure VR system: 2D glasses with surround‐sound headset (HappyMed) Procedure duration 46.2, (SD 15.3) min Side effects: *n* = 3 (5%) cybersickness	**Control:** Usual care (no VR): no sedation, analgesia given when required. Procedure duration 43.6, (SD 10.6) min	Peri‐procedural anxiety measured post‐procedure via STAI Post‐procedural pain via 10‐point VAS Patient's perceived procedure duration Satisfaction with VR	Lower peri‐procedural anxiety in VR group (31.5, SD 13.4) than control (38.5, SD 19.2) *p* < 0.05^b^ Post‐procedural pain not different between post‐VR (3.6, SD 2.8) and control (4.2, SD 3.4), P > 0.05^b^ Lower perceived procedure duration in VR group (60.1, SD 32.3 min) than control (73, SD 32.4 min) *p* = 0.04^c^ 88.9% recommended VR glasses for TAVI procedures
Peri‐procedure	Roxburgh et al. (2021), France	Non‐randomised controlled trial (consecutive cohort)	Total *n* = 99 **VR** *n* = 48; Age: 63 years (SD 10.9 years); 33% F **Control** *n* = 51; Age: 64.5 years (SD 10.4 years); 24% F	Interventional: Cryoballoon ablation for AF	VR‐based distraction plus usual care Delivered peri‐procedure with 5 min cardiac coherence breathing before VR VR head‐mounted display audio visual (Deepsen) Unknown duration Tolerance: 14.6% removed VR during procedure Side effects: *n* = 3 (6%) cybersickness	**Control:** usual care (consecutive cohort of patients who receivedroutine AF cryoablation in the 3 months prior to the study usingthe standard analgesia)	Peri‐procedural overall and max pain measured 45‐min post‐procedure via 0–10 point VAS Peri‐procedural comfort measured 45‐min post‐procedure via 0–10 point VAS	Lower overall peri‐procedural pain in VR group (3.5, SD 1.5) than control (4.3, SD 1.6) *p* = 0.004^c^ Lower max peri‐procedural pain in VR group (5.1, SD 1.9) than control (6.1, SD 2.0) *p* = 0.003^c^ Higher comfort in VR group (7.5, SD 1.6) than control (6.8, SD 1.7) p = 0.03^c^
Peri‐procedure	Zablah et al. (2022), USA	Self controlled case series	Total *n* = 3 14–15 years old; *n* = 2 male, *n* = 1 F	Interventional: Diagnostic cardiac catheterisation	VR‐based distraction plus analgesia Delivered peri‐procedure VR head‐mounted display (Oculus Go) with hand‐operated console (no audio details) Unknown duration Side effects: none	All 3 patients had procedure without VR before having procedure with VR. Analgesia given	Peri‐procedural anxiety measured post‐procedure via 10‐point VAS Peri‐procedural pain measured post‐procedure via 10‐point VAS Recommend VR via 10‐point scale	Peri‐procedural anxiety: 3/10 or less by all participants post‐VR Peri‐procedural pain: 2/10 or less by all participants post‐VR Recommend VR: 9/10 or higher by all participants
**Studies evaluating *VR‐based exercise rehabilitation* interventions**								
Post‐procedure	Cacau et al. (2013), Brazil	RCT	Total *n* = 60 **VR** *n* = 30; Age 49.2 years (SEM 2.6 years); 57% F **Control** *n* = 30; Age 52 years (SEM 2.4 years); 47% F	Surgical: CABG or SVR	VR‐based exercise rehabilitation Delivered during recovery from surgery within hospital VR headset not reported Usual care physical therapy with exercise performed using VR twice a day until discharge home Side effects: not reported	**Control:** usual care physical therapy not using VR	Generic life quality evaluation via Nottingham Health Profile (NHP) on 1st, 3rd post‐procedure day and discharge day Physical function: Walking capacity via 30 m 6MWT at discharge Motor and cognitive functional performance via Functional Independent Measure (FIM)	NHP pain sub‐score: change from pre to discharge was greater (improved pain levels) in VR than control *p* < 0.05^c^ Greater improvement in NHP energy sub‐score with VR than control at 1st day post‐op; *p* < 0.05^c^ NHP total score not different Higher 6MWT in VR group (320 m, SEM 19 m) than control (264 m, SEM 15 m) *p* < 0.05^c^ Higher motor FIM score in VR group than control on 1^st^ day post‐op; *p* < 0.05^c^ Cognitive FIM score not different between groups at any time
Post‐procedure	Lima et al. (2020), Brazil	RCT	Total *n* = 56 **VR** *n* = 31; Age 54 years (SD 8 years); 32% F **Control** *n* = 25; Age 51 years (SD 10 years); 32% F	Surgical: CABG	VR‐based exercise rehabilitation Delivered during recovery from surgery within hospital VR (headset not reported) via XBOX 360 platform and Kinect electronic device displayed on TV Performed twice a day for 5 days in addition to usual care Side effects: not reported	**Control:** usual care conventional physiotherapy (free active kinesiotherapy, cycle ergometry, ambulation and expansive ventilatory patterns) twice a day for 5 days	Pulmonary function via the Maximum Inspiratory Pressure (MIP), Maximum Expiratory Pressure (MEP), Peak Expiratory Flow (PEF) and Vital Capacity (VC) Physical function via the Timed Up and Go (TUG) test and FIM questionnaire	Higher post‐procedure (5 days) MIP, MEP, PEF with VR than control *p* < 0.001^c^ VC not different between groups Improved post‐procedure (5 days) TUG in VR group (10, SD 2 s) than control (22, SD 9 s) *p* < 0.001^c^ Higher post‐procedure (5 days) FIM score with VR (120, SD 3) than control (112, SD 5) *p* < 0.001^c^
Post‐procedure	Ribeiro et al. (2021), Brazil	RCT	Total *n* = 48 **VR** *n* = 17, Age 62.1 years (SD 9 years); 41.2% F **Early mobilisation** *n* = 15, Age 58.3 years (SD 7.7 years), 13.3% F **Control** *n* = 16; Age 60.3 years (SD 8.3 years); 25% F	Surgical: CABG	VR‐based exercise rehabilitation Delivered during recovery from surgery within hospital VR (headset not reported) via Nintendo Wii games Performed on 1^st^ to 3^rd^ day post‐procedure plus usual care physiotherapy and cycle ergometer exercise Side effects: not reported	**Control:** usual care respiratory physio; foot and ankle exercises **Early mobilisation Group (EMG):** usual care plus cycle ergometer and ambulation	HR variability pre‐ and 4^th^ day post‐procedure (PO4) recorded for 10 min period following a 10 min period of supine rest	Several indices of HR variability lower in control group than VR and EMG, indicating greater parasympathetic influence and improved cardiac autonomic modulation (*p* < 0.05^c^)

*Note:* Mean, standard deviation (SD) unless stated otherwise.

Abbreviations: 6MWT: 6‐min walk test; AF: atrial fibrilation; BHF: British Heart Foundation (charity organisation); BP: blood pressure; CABG: coronary artery bypass graft; CSQ‐8: client satisfaction questionnaire‐8; Dr.: doctor; EP: electrophysiological; F: female; HR: heart rate; ICU: intensive care unit; IQR: interquartile range; MEP: maximum expiratory pressure; MIP: maximum inspiratory pressure; NS: non‐significant; PEF: peak expiratory flow; RB: rhythmic breathing; RCT: randomised controlled trial; RR: respiratory rate; SAVR: surgical aortic valve replacement; SEM: standard error mean; STAI: state–trait anxiety inventory; SVR: surgical valve replacement; TAA: thoracic aortic aneurysm; TAVI: transcatheter aortic valve implantation; TUG: timed up and go; UC: usual care; VAS: visual analogue scale; VC: vital capacity; VR: virtual reality.

^a^ Study included in meta‐analysis.

^b^ Indicates results from meta‐analysis.

^c^ Indicates statistical significance reported in the study.

### 3.3 Characteristics of VR Interventions

All studies included in this review used VR rather than other types of XR intervention. Four types of VR intervention were delivered: VR‐facilitated education, VR‐based relaxation, VR‐based distraction and VR‐based exercise rehabilitation. The timing of VR intervention delivery varied; nine studies delivered the VR intervention before cardiac surgery (Grab et al. 2023; Hendricks et al. 2020) or an interventional procedure (Aardoom et al. 2022; Chang et al. 2020, 2021; Keshvari et al. 2021; Morgan et al. 2021; Oudkerk Pool et al. 2022; Pouryousef et al. 2020), seven during a cardiac interventional procedure while the participants were conscious (Bruno et al. 2020; Coulibaly et al. 2022; Laghlam et al. 2021; Lanquetuit et al. 2022; Lind et al. 2023; Roxburgh et al. 2021; Zablah et al. 2022), three after cardiac surgery (Cacau et al. 2013; Lima et al. 2020; Ribeiro et al. 2021) and two after either cardiac surgical or interventional procedures (Mosso‐Vázquez et al. 2014, 2013). One study delivered VR twice, before and after cardiac surgery (Rousseaux et al. 2022).

VR interventions were predominately delivered in hospital settings except in one study, in which 50% of participants received VR in the hospital, and 50% at home ahead of the procedure (Aardoom et al. 2022). The amount of detail provided about VR interventions (technical set‐up) and control groups varied. Technical details about the VR headset were provided by 15 (68%) studies.

#### 3.3.1 VR‐Facilitated Education Interventions

VR‐facilitated education was evaluated in six studies and delivered pre‐cardiac surgery with average duration of 22 min (Grab et al. 2023) or pre‐cardiac interventional procedure with duration ranging between 3 min and 22 min (Aardoom et al. 2022; Chang et al. 2020, 2021; Morgan et al. 2021; Oudkerk Pool et al. 2022). VR‐facilitated education interventions involved participants using VR headsets to watch and learn about the procedure. Procedural details with models of the heart were shown in one VR intervention with a verbal explanation provided in real‐time with the cardiologist (Grab et al. 2023). In the other five studies, the patient journey on the day of the procedure, including details of the procedure, was shown in VR videos with simultaneous audio included in four studies (Aardoom et al. 2022; Chang et al. 2020, 2021; Morgan et al. 2021; Oudkerk Pool et al. 2022). One study did not provide audio details of their VR intervention (Oudkerk Pool et al. 2022). Interaction and immersion details were not always reported. Three studies reported that participants could select the content during the VR intervention (Aardoom et al. 2022; Chang et al. 2020, 2021) and four studies reported the use of 360° immersion (Aardoom et al. 2022; Chang et al. 2020, 2021; Oudkerk Pool et al. 2022).

#### 3.3.2 VR‐Based Relaxation Interventions

VR‐based relaxation interventions were evaluated in nine studies and were delivered pre‐cardiac interventional procedure in two studies (Keshvari et al. 2021; Pouryousef et al. 2020), peri‐cardiac interventional procedure in four studies (Bruno et al. 2020; Coulibaly et al. 2022; Laghlam et al. 2021; Lanquetuit et al. 2022), post‐procedure (surgical or interventional procedure) in two studies (Mosso‐Vázquez et al. 2014, 2013), and pre‐ and post‐cardiac surgery in one study (Rousseaux et al. 2022). Duration ranged between 5 min and approximately 60 min. One study reported limited details of the VR intervention (Pouryousef et al. 2020). In the remaining studies, all the VR interventions included scenes of the natural environment (e.g., mountains, forest and ocean). One study involved 3D audio‐visual immersion with interactivity (Coulibaly et al. 2022). Four other studies involved 3D/360° immersive VR interventions but details on audio and interactive content varied (Bruno et al. 2020; Keshvari et al. 2021; Laghlam et al. 2021; Rousseaux et al. 2022). Immersive and interactive details were absent from three other studies (Lanquetuit et al. 2022; Mosso‐Vázquez et al. 2014, 2013).

#### 3.3.3 VR‐Based Distraction Interventions

VR interventions designed to distract users were evaluated in four studies and delivered pre‐cardiac surgery in one study with duration of 20 min (Hendricks et al. 2020) and peri‐interventional cardiac procedure in three studies for the duration of the procedure, which was an average of 46 min in one study (Lind et al. 2023) and not reported in the remaining two studies (Roxburgh et al. 2021; Zablah et al. 2022). Distraction content included 2D films (Lind et al. 2023), 3D computer‐simulated audio‐visual scenarios with interaction with environment (Roxburgh et al. 2021), 3D environment/movie (unknown audio) with interaction (Zablah et al. 2022) and 360° immersive games with audio and interaction (Hendricks et al. 2020).

#### 3.3.4 VR‐Based Exercise Rehabilitation Interventions

VR‐based exercise rehabilitation was evaluated in the postoperative period following cardiac surgery in three studies (Cacau et al. 2013; Lima et al. 2020; Ribeiro et al. 2021). In addition to usual care physical therapy, participants used VR to perform exercise or virtual sports games for either twice per day until discharge (Cacau et al. 2013), twice per day for 5 days (Lima et al. 2020) or daily for the first three postsurgical days (Ribeiro et al. 2021). Characteristics of the VR headset was not reported; therefore, the level of immersion was unknown.

### 3.4 Quality of Studies

**FIGURE 2:**
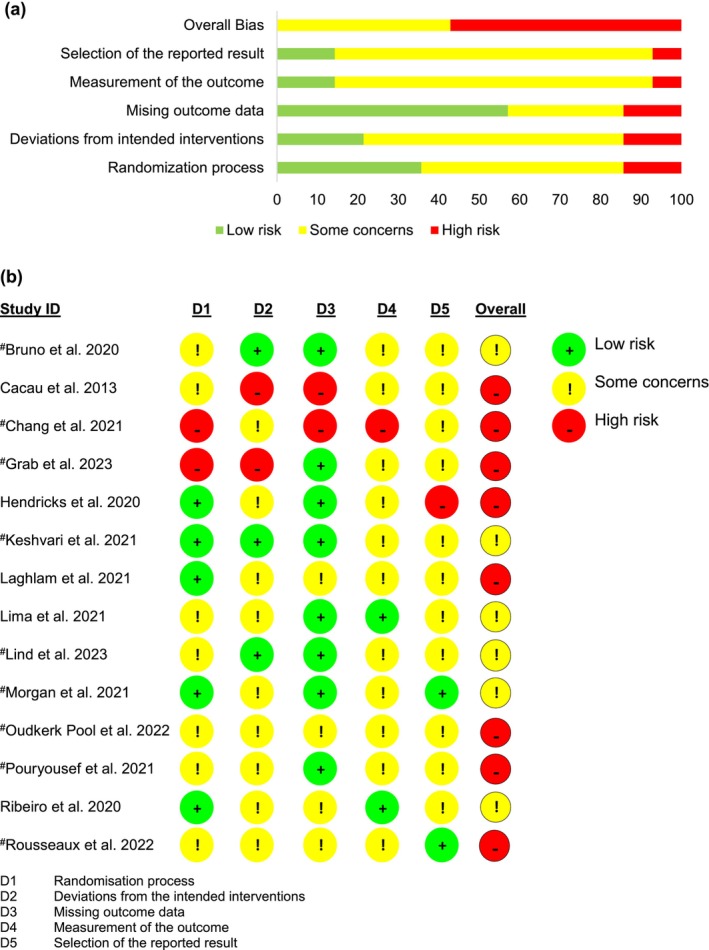
Risk of bias: (a) summary and (b) assessment of individual studies. ^#^Indicates study included in meta‐analysis. [Colour figure can be viewed at wileyonlinelibrary.com]

Figure 2 summarises the risk of bias of the 14 randomised trials assessed using the ROB2 tool. Eight (57%) studies were rated as having a ‘high’ overall risk of bias and none as having a ‘low’ risk of bias. To be rated as ‘low risk’ for domain 5 of the ROB2 tool, a full study protocol/analysis plan should be available; two studies fulfilled this criterion (Morgan et al. 2021; Rousseaux et al. 2022). In the quality assessment of the eight non‐randomised studies, five were rated as ‘poor quality’ (Aardoom et al. 2022; Chang et al. 2020; Mosso‐Vázquez et al. 2014, 2013; Zablah et al. 2022), one as ‘some concerns’ (Lanquetuit et al. 2022) and two as ‘good quality’ (Coulibaly et al. 2022; Roxburgh et al. 2021).

### 3.5 Results of Meta‐Analyses

#### 3.5.1 Pre‐Procedural Anxiety

Nine studies measured the effect of VR interventions delivered before the cardiac procedure on pre‐procedural anxiety. Two studies were ineligible for inclusion in the meta‐analysis due to study design (Chang et al. 2020) and incomparable control group (Hendricks et al. 2020), leaving seven RCTs included in the meta‐analysis (Chang et al. 2021; Grab et al. 2023; Keshvari et al. 2021; Morgan et al. 2021; Oudkerk Pool et al. 2022; Pouryousef et al. 2020; Rousseaux et al. 2022). The sample size of these seven studies ranged from 33 (Chang et al. 2021) to 100 (Rousseaux et al. 2022). Participants were scheduled for cardiac catheterisation/angiography (Keshvari et al. 2021; Morgan et al. 2021; Pouryousef et al. 2020), atrial septal defect/patent foramen ovale closure (Oudkerk Pool et al. 2022), catheter ablation (Chang et al. 2021) or cardiac surgery (Grab et al. 2023; Rousseaux et al. 2022). Interventions were either VR‐facilitated education (Chang et al. 2021; Grab et al. 2023; Morgan et al. 2021; Oudkerk Pool et al. 2022) or VR‐based relaxation (Keshvari et al. 2021; Pouryousef et al. 2020; Rousseaux et al. 2022). Control groups in studies evaluating VR‐facilitated education interventions were usual care, which consisted of paper‐based educational materials and explanations from health professionals (Chang et al. 2021; Grab et al. 2023; Oudkerk Pool et al. 2022) plus an online video in one study (Morgan et al. 2021). Usual care control groups in studies evaluating VR‐based relaxation interventions were the absence of relaxation techniques or VR (Keshvari et al. 2021; Pouryousef et al. 2020; Rousseaux et al. 2022).

**FIGURE 3:**
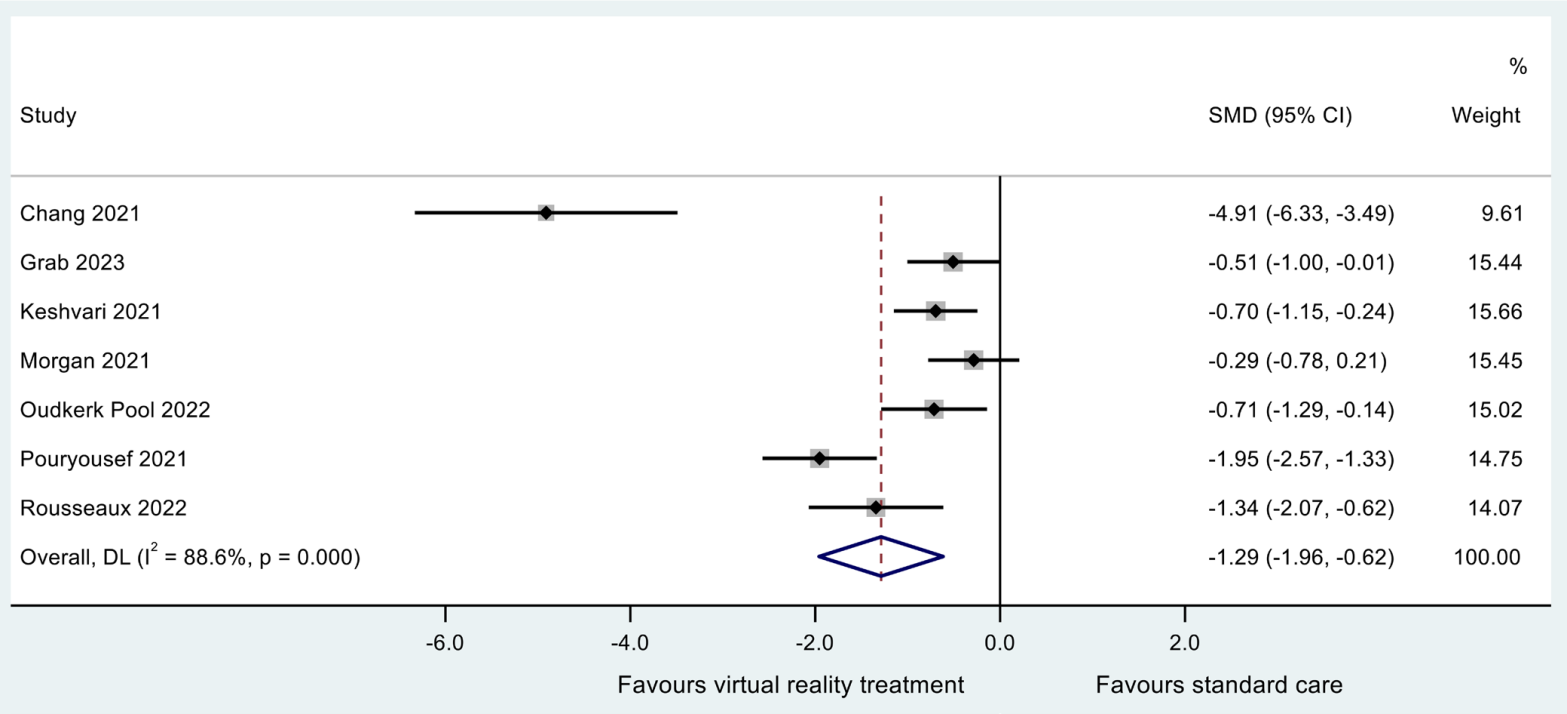
Forest plot for meta‐analysis of pre‐procedural anxiety. [Colour figure can be viewed at wileyonlinelibrary.com]

**FIGURE 4:**
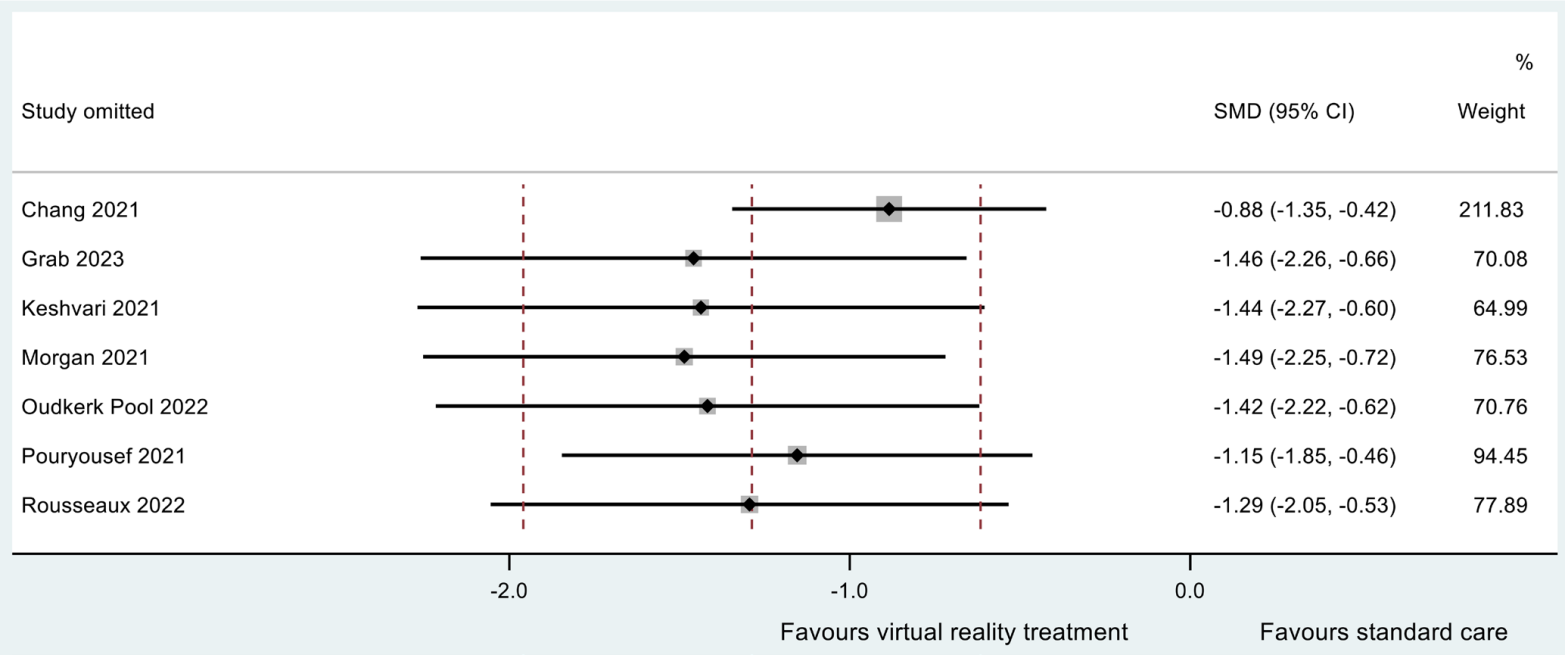
Influence plot for meta‐analysis of pre‐procedural anxiety. [Colour figure can be viewed at wileyonlinelibrary.com]

Six RCTs reported a significant decrease (*p* < 0.05) in pre‐procedural anxiety compared to standard care (Chang et al. 2021; Grab et al. 2023; Keshvari et al. 2021; Oudkerk Pool et al. 2022; Pouryousef et al. 2020; Rousseaux et al. 2022). One RCT reported a non‐significant decrease in pre‐procedural anxiety compared to standard care (Morgan et al. 2021). The synthesised estimate for the standardised mean difference (VR group minus standard care group) was −1.29 (95% CI ‐1.96, −0.62); that is, pre‐procedural anxiety was lower in those receiving VR. A *Z*‐test of the standardised mean difference revealed strong evidence at the 5% significance level for a non‐zero effect (*Z* = 3.76; *p* = 0.001). Cochran's chi‐square test for heterogeneity revealed evidence for statistical heterogeneity (*χ* ^2^_(6)_ = 52.7; *p* < 0.001). The *I* ^2^ statistic (the proportion of total variation in effect estimate due to between‐study heterogeneity) was 95.5%. The *τ* ^2^ statistic was 0.696. The data are summarised in a forest plot (Figure 3). A sensitivity study revealed no individual study to be exerting excessive influence on the analysis, with all point estimates of the omitted analyses lying within the 95% CI associated with the estimate of the combined analysis (Figure 4).

#### 3.5.2 Peri‐Procedural Anxiety

Five studies measured the effect of VR interventions delivered during the cardiac procedure on peri‐procedural anxiety. Three studies were ineligible for inclusion in the meta‐analysis due to study design (absence of, or incomparable control group) (Laghlam et al. 2021; Lanquetuit et al. 2022; Zablah et al. 2022), leaving two RCTs included in the meta‐analysis with sample sizes of 32 (Bruno et al. 2020) and 117 (Lind et al. 2023). Participants in both RCTs were undergoing TAVI and received either VR‐based relaxation (Bruno et al. 2020) or distraction interventions (Lind et al. 2023). Peri‐procedural anxiety was measured retrospectively either immediately after TAVI via the State–Trait Anxiety Inventory (Lind et al. 2023) or one‐day post‐TAVI via a 10‐point visual analogue scale (Bruno et al. 2020). The control groups in both were usual care no VR intervention.

**FIGURE 5:**
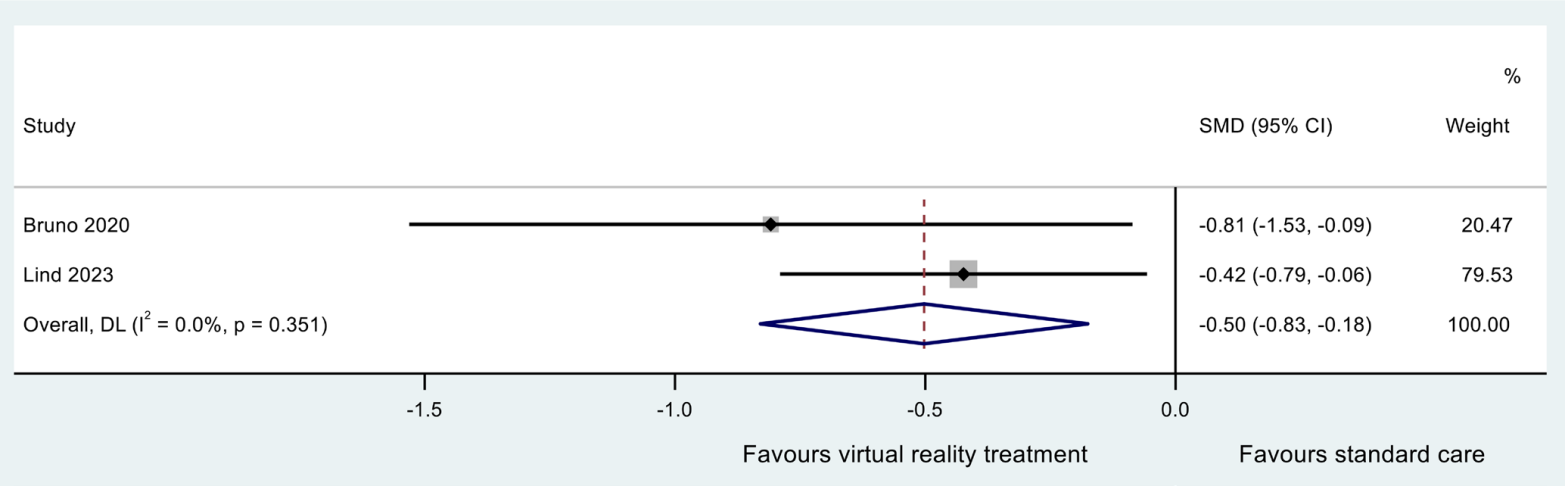
Forest plot for meta‐analysis of peri‐procedural anxiety (VR delivered during procedure). [Colour figure can be viewed at wileyonlinelibrary.com]

Both RCTs included in the meta‐analysis reported a significant decrease (*p* < 0.05) in peri‐procedural anxiety compared to standard care (Bruno et al. 2020; Lind et al. 2023). The synthesised estimate for the standardised mean difference (VR group minus standard care group) was −0.50 (95% CI −0.83, −0.18); that is, peri‐procedural anxiety was lower in those receiving VR during the procedure. A *Z*‐test of the standardised mean difference revealed strong evidence at the 5% significance level for a non‐zero effect (*Z* = 3.01; *p* = 0.003). Cochran's chi‐square test for heterogeneity revealed no evidence for statistical heterogeneity (*χ* ^2^_(1)_ = 0.87; *p* = 0.351). The *I* ^2^ statistic (proportion of total variation in effect estimate due to between‐study heterogeneity) and *τ* ^2^ statistics were approximately zero. The data are summarised in a forest plot (Figure 5). Due to the low number of included studies, sensitivity analyses and tests for small study effects were not conducted.

#### 3.5.3 Peri/Post‐Procedural Pain Levels

Eleven studies measured the effect of VR interventions delivered peri‐, or post‐cardiac procedure on patient‐reported procedural or postoperative pain levels. Seven studies were ineligible for inclusion in the meta‐analysis due to study design (absence of, or incomparable control group) (Laghlam et al. 2021; Lanquetuit et al. 2022; Mosso‐Vázquez et al. 2014, 2013; Roxburgh et al. 2021; Zablah et al. 2022) or incomparable outcome measure (Cacau et al. 2013). Three RCTs (Bruno et al. 2020; Lind et al. 2023; Rousseaux et al. 2022) and one non‐randomised controlled trial (Coulibaly et al. 2022) were included in the meta‐analysis with sample size ranging from 32 to 117. VR‐facilitated relaxation or distraction interventions were delivered during TAVI (Bruno et al. 2020; Lind et al. 2023), cardiac pacing (Coulibaly et al. 2022) or post‐cardiac surgery (Rousseaux et al. 2022). The control groups in all studies were usual care with no VR intervention or relaxation techniques.

**FIGURE 6:**
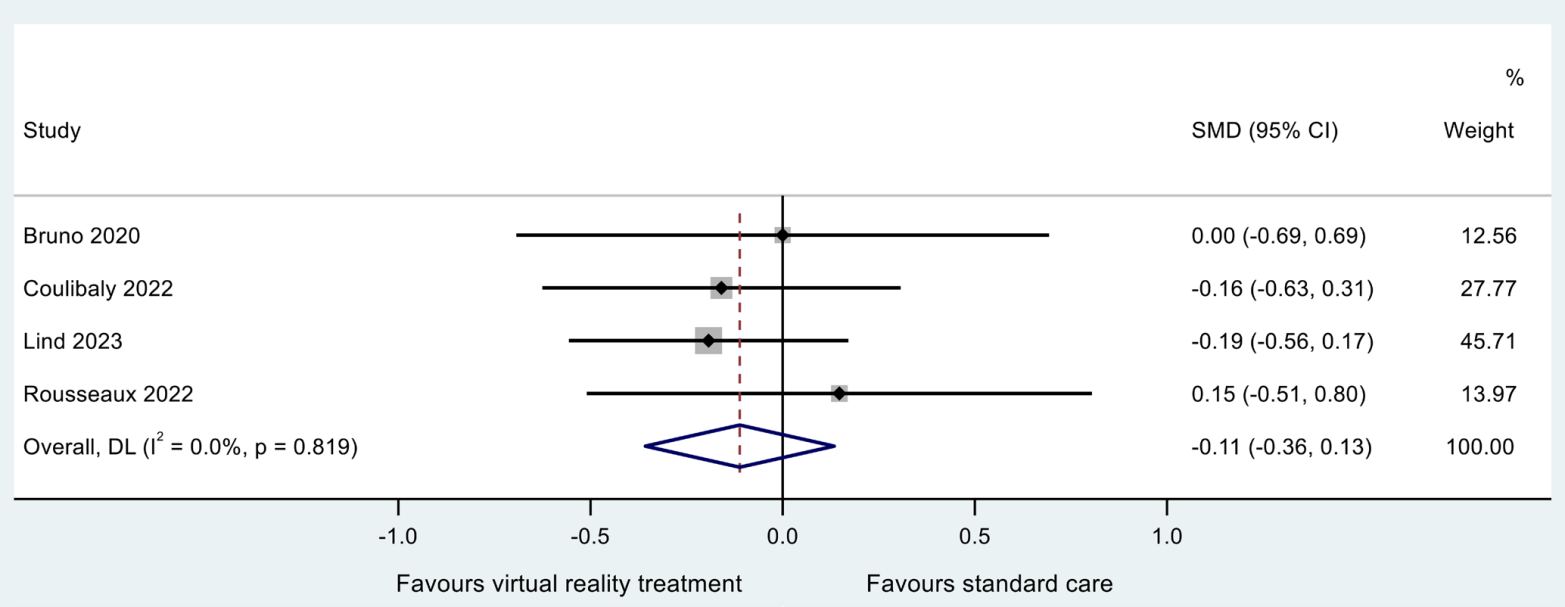
Forest plot for meta‐analysis of post‐procedural pain (VR delivered during or after the procedure). [Colour figure can be viewed at wileyonlinelibrary.com]

**FIGURE 7:**
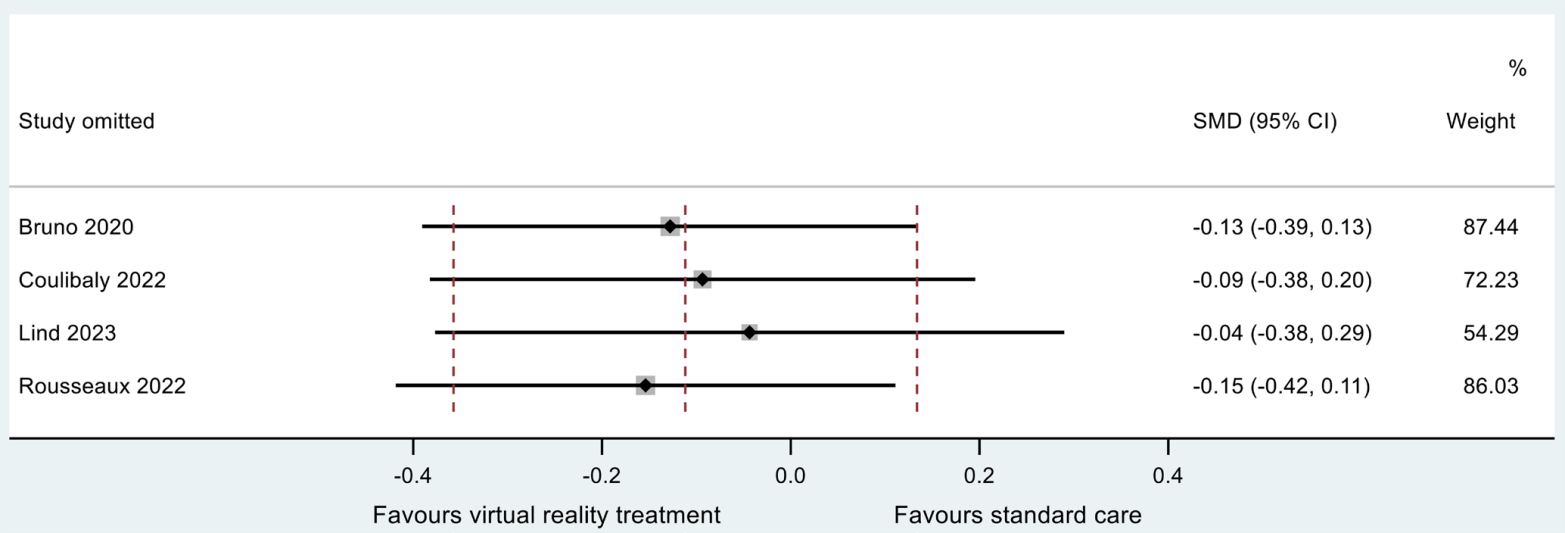
Influence plot for meta‐analysis of post‐procedural pain (VR delivered during or after the procedure). [Colour figure can be viewed at wileyonlinelibrary.com]

Two studies reported a non‐significant decrease (*p* > 0.05) in post‐procedural pain of those receiving VR interventions compared to standard care (Coulibaly et al. 2022; Lind et al. 2023). One study reported a non‐significant increase in post‐procedural pain of those receiving VR interventions compared to standard care (Rousseaux et al. 2022). One study reported no significant difference in post‐procedural pain between those receiving VR interventions and those receiving standard care (Bruno et al. 2020). The synthesised estimate for the standardised mean difference (VR group minus standard care group) was −0.11 (95% CI −0.36, 0.13); that is, pain was lower in those receiving VR care during or after the procedure. A *Z*‐test of the standardised mean difference revealed no evidence at the 5% significance level for a non‐zero effect (*Z* = 0.892; *p* = 0.372). Cochran's chi‐square test for heterogeneity revealed no evidence for statistical heterogeneity (*χ* ^2^_(3)_ = 0.93; *p* = 0.819). The *I* ^2^ and *τ* ^2^ statistic for this analysis were zero, indicating negligible proportion of variation across studies ascribed to heterogeneity. The data are summarised in a forest plot (Figure 6). A sensitivity study revealed no individual study to be exerting excessive influence on the analysis, with all point estimates of the omitted analyses lying within the 95% CI associated with the estimate of the combined analysis. Estimates and associated CIs are plotted on an influence plot (Figure 7).

### 3.6 Synthesis Without Meta‐Analysis

#### 3.6.1 Emotional Functioning and Wellbeing

In addition to the effect of VR interventions on anxiety levels reported in the meta‐analyses, other outcomes related to emotional functioning and wellbeing were evaluated in five studies that reported contrasting results. In a randomised trial, participants who received VR‐based distraction felt significantly less tense/strained and calmer, but conversely felt less steady compared to those who received a non‐VR‐based game before cardiac surgery (Hendricks et al. 2020). Two studies evaluated VR‐facilitated education before cardiac catheterisation. In a single group post‐test study design, 88% of participants reported a reduction in negative psychological emotions following VR‐facilitated education delivered pre‐procedure (Aardoom et al. 2022). However, in an RCT, feelings of concern and helplessness during cardiac catheterisation were no different between participants that received VR‐facilitated education pre‐procedure compared with usual care (Morgan et al. 2021). Another RCT found no differences in patient‐reported levels of fatigue and relaxation following VR‐facilitated relaxation delivered before and after cardiac surgery, compared with comparative groups (usual care or hypnosis) (Rousseaux et al. 2022). In contrast, patient‐reported energy levels were significantly higher following VR‐facilitated exercise rehabilitation delivered post‐cardiac surgery compared to usual care (Cacau et al. 2013). However, the difference between groups in this RCT was not sustained after the first postoperative day.

#### 3.6.2 Cognitive Functioning

The effect of VR interventions on outcomes related to ‘cognitive functioning’ were measured via the patient‐reported functional independence measure (cognitive function score) in one study (Cacau et al. 2013) and by patient knowledge and self‐reported understanding questionnaires in five studies (Aardoom et al. 2022; Chang et al. 2020, 2021; Grab et al. 2023; Morgan et al. 2021). VR‐based exercise rehabilitation delivered following cardiac surgery in one RCT had no effect on patient‐reported cognitive function compared to usual care, with high scores reported in both groups at pre‐ and postoperative days (Cacau et al. 2013). Patient knowledge was evaluated following VR‐facilitated education in two RCTs which found significantly greater knowledge scores post‐VR than usual care (education with paper‐based materials) in relation to AF ablation procedural and after care knowledge (Chang et al. 2021) and CABG and SAVR procedural understanding (Grab et al. 2023). In the latter study, higher pre‐procedural knowledge was significantly associated with lower pre‐procedural anxiety (Grab et al. 2023). Similarly, results from a non‐controlled study showed significantly higher knowledge scores about AF ablation post‐VR‐facilitated education compared to before receiving the intervention (Chang et al. 2020). In relation to self‐reported understanding, an RCT found that participants in the VR group felt significantly better informed about how the coronary angiogram was performed compared to the usual care group, but there was no difference between groups in their perceived understanding of why they were having the procedure (Morgan et al. 2021). Usual care in this study included written and verbal information plus an online informational video. Participants in the VR group also had access to the same written and verbal patient information as the control group. Ratings for receiving sufficient information pre‐procedure were high in both groups (Morgan et al. 2021). Similarly, in a post‐test study design, participants felt better informed and prepared about cardiac catheterisation post‐VR‐facilitated education (Aardoom et al. 2022).

### 3.7 Physical Functioning

The effect of VR interventions on outcomes related to ‘physical functioning’ were evaluated in two RCTs via patient‐reported and objective measures of physical performance (Cacau et al. 2013; Lima et al. 2020). In both RCTs, VR‐based exercise rehabilitation delivered following cardiac surgery significantly improved the ‘motor subscale’ of the functional independence measure and walking capacity of participants compared to those who received usual care (Cacau et al. 2013; Lima et al. 2020).

### 3.8 Delivery of Care

Fifteen studies evaluated the effect of VR interventions on a range of outcomes linked to care delivery: levels of self‐reported physical comfort/discomfort during or after a cardiac procedure or surgery, unmet needs for procedural information, perceived duration of procedure and satisfaction with the cardiac procedure and/or healthcare team. Outcomes about participants level of tolerance to VR and their self‐reported satisfaction with VR were also included.

Patient‐reported levels of physical comfort/discomfort experienced during and after cardiac procedures were measured in two non‐randomised controlled trials (Coulibaly et al. 2022; Roxburgh et al. 2021) and two non‐controlled studies (Lanquetuit et al. 2022; Mosso‐Vázquez et al. 2013). Contrasting findings were reported, which might be explained by the different cardiac procedures or timing of VR interventions. Levels of comfort with AF catheter ablation were rated higher in the group of participants who received VR‐based distraction during the procedure than those in the control group who received usual care (Roxburgh et al. 2021). However, comfort levels were significantly worse 10‐mins post‐cardiac pacing procedure, and at discharge (unknown time post‐procedure), in the intervention group who received VR‐based relaxation during the procedure, compared to those in the usual care group (Coulibaly et al. 2022). In a non‐controlled study, low levels of discomfort were reported during interventional cardiac procedures with VR‐based relaxation (Lanquetuit et al. 2022). When VR‐based relaxation was delivered after a cardiac procedure or surgery, one study found that 95% of participants reported less discomfort post‐VR than pre‐VR (Mosso‐Vázquez et al. 2013).

Participants ability to tolerate VR interventions was reported by 12 studies that evaluated either VR‐facilitated education (Aardoom et al. 2022; Chang et al. 2020; Grab et al. 2023), VR‐based relaxation (Bruno et al. 2020; Coulibaly et al. 2022; Laghlam et al. 2021; Lanquetuit et al. 2022; Mosso‐Vázquez et al. 2014, 2013) or VR‐based distraction (Lind et al. 2023; Roxburgh et al. 2021; Zablah et al. 2022). In three of these studies, participants reported no side effects associated with using VR interventions; (VR‐facilitated education pre‐interventional cardiac procedures (Aardoom et al. 2022; Chang et al. 2020), or VR‐based distraction delivered during interventional cardiac procedures (Zablah et al. 2022). In contrast, in seven studies, 3%–14% of participants using VR reported side effects of cybersickness, characterised by nausea, vertigo and/or headache (Grab et al. 2023; Laghlam et al. 2021; Lanquetuit et al. 2022; Lind et al. 2023; Mosso‐Vázquez et al. 2014, 2013; Roxburgh et al. 2021). In the remaining two studies, 30.5% of participants removed the VR headset during cardiac pacing procedures (Coulibaly et al. 2022) and 62.5% during TAVI (Bruno et al. 2020), due to discomfort or heat. In studies evaluating VR‐based exercise rehabilitation, participant ability to tolerate VR interventions was not measured.

Ten studies evaluated participants levels of satisfaction with VR interventions/and or the cardiac procedure (Aardoom et al. 2022; Bruno et al. 2020; Chang et al. 2020, 2021; Grab et al. 2023; Laghlam et al. 2021; Lanquetuit et al. 2022; Lind et al. 2023; Morgan et al. 2021; Zablah et al. 2022). Although results were predominantly positive, three of the studies reported contrasting results. Satisfaction with VR‐facilitated education delivered pre‐cardiac procedure was significantly higher than paper‐based education (Chang et al. 2021), but not significantly different when compared with a combination of paper, verbal and video‐based information (Morgan et al. 2021). When VR was delivered during a cardiac procedure, satisfaction with the procedure was significantly less than the group who received Kalinox (mix of oxygen and nitric oxide) (Laghlam et al. 2021).

In regard to other related outcomes, participants need for information did not differ between participants who received VR‐facilitated education or usual care before transcutaneous closure procedures (Oudkerk Pool et al. 2022). The implementation of VR‐based distraction during TAVI influenced participants' perceptions about the duration of the procedure. The VR group perceived the TAVI procedure to be, on average, 13 min less than the usual care group, despite there being only 3 minutes average difference in the actual duration (Lind et al. 2023).

### 3.9 Physiological Outcomes

Five RCTs (Bruno et al. 2020; Keshvari et al. 2021; Lima et al. 2020; Ribeiro et al. 2021; Rousseaux et al. 2022) and two pre‐post‐test studies (Mosso‐Vázquez et al. 2014, 2013) measured the effects of VR interventions on haemodynamics, or pulmonary function. In two RCTs, VR‐based exercise rehabilitation delivered following cardiac surgery significantly improved pulmonary function (Lima et al. 2020) and heart rate variability (Ribeiro et al. 2021). Five studies evaluated the effect of VR‐based relaxation on haemodynamic parameters with inconsistent results. One RCT showed that 5 minutes of VR‐based relaxation, delivered before coronary angiography, significantly lowered both heart rate compared with control, and systolic blood pressure (on average of 11 mmHg), compared to pre‐VR (Keshvari et al. 2021). However, two other RCTs examining the effect of VR on blood pressure reported no significant difference at any stage of patient care, when compared to usual care, or an experimental arm delivering hypnosis (Bruno et al. 2020; Rousseaux et al. 2022). In two pre–post‐test studies, 30 min of VR‐based relaxation delivered after interventional cardiac procedures lowered respiratory rate and blood pressure in over 50% of participants with inconsistent effects on heart rate (Mosso‐Vázquez et al. 2014, 2013).

## 4 Discussion

### 4.1 Summary of Main Findings

This robust systematic review and meta‐analysis of the international literature is the first to evaluate how XR interventions may benefit patients, before, during or after elective cardiac surgical and interventional procedures. VR interventions were the only XR technology identified in this context. Results from the meta‐analyses suggest that VR interventions are effective in reducing patient anxiety levels before, and during, elective cardiac and surgical procedures, but fail to significantly reduce pain levels during or after these procedures. The accompanying narrative synthesis details the potential benefits of VR on patient outcomes outlined in the COMET typology.

### 4.2 VR Interventions and Patient Anxiety

High levels of anxiety experienced before, or during, cardiac related procedures can negatively impact on patient outcomes by inducing endothelial dysfunction (Henein et al. 2022), amplifying perceived pain levels and slowing recovery (Liblik et al. 2023; Tadesse et al. 2022; Trotter, Gallagher, and Donoghue 2011). Fear about complications (Caldwell et al. 2007), a lack of procedural knowledge (Hernández‐Palazón et al. 2018) and lower patient educational levels (Delewi et al. 2017) are all factors associated with higher levels of patient anxiety.

Our meta‐analyses demonstrated that VR interventions have a significant large effect in the reduction of pre‐procedural anxiety levels in cardiac patients undergoing cardiac catheterisation/angiography, atrial septal defect/patent foramen ovale closure, catheter ablation or cardiac surgery, and a significant moderate effect in reducing anxiety levels during TAVI procedures with and without conscious sedation. Our findings are supported by those reported in similar meta‐analyses of VR interventions implemented across medical procedures and surgeries (Gao, Wang, and Liu 2023; Kodvavi et al. 2023). The use of VR interventions warrants further research as they have the potential to improve the patient care experience and improve clinical outcomes by reducing patient anxiety.

The implementation of VR interventions for people undergoing TAVI shows significant potential. Particularly as the number of TAVI procedures have increased dramatically from the first implantation in 2002, to approximately 300,000 annually worldwide (Patterson et al. 2020). TAVI is an alternative to open heart surgery, which relieves life‐limiting symptoms associated with degenerative aortic stenosis. It is typically performed while the patient is conscious and can last up to 2 h so can be unpleasant. Moreover, sedation offered to patients, can induce delirium in older adults and should be minimised where possible (American Geriatrics Society Expert Panel on Postoperative Delirium in Older Adults 2015). The beneficial effects of VR on reducing anxiety during TAVI, with or without conscious sedation, are an original finding that can directly inform care provision. Effective interventions that reduce anxiety and the use of sedatives during TAVI, such as VR, offer an alternative non‐pharmacological approach that warrants further research.

Although VR interventions delivered during TAVI reduced anxiety (Bruno et al. 2020; Lind et al. 2023), our findings did not indicate an effect on pain levels during or after the procedure. This novel finding suggests that greater relaxation or distraction might be required to reduce pain levels compared to the amount that is needed to relieve anxiety during TAVI. Future research is needed to test this hypothesis.

### 4.3 VR Interventions and Pain Management

Patients may experience temporary pain and discomfort, during and after cardiac interventional procedures (Brogiene et al. 2022) or after cardiac surgery (Xiao et al. 2023). Analgesia offer relief but many hospital inpatients report that their pain could have been more effectively managed (Care Quality Commission 2023). VR interventions have potential as an adjunct to analgesia to offer effective pain relief for individuals during labour, endoscopic procedures, dressing changes, needle‐related procedures or after orthopaedic and abdominal surgery (Boyce et al. 2023; Huang et al. 2022; Viderman et al. 2023; Xu et al. 2022).

Our meta‐analysis indicated that VR‐based relaxation and distraction interventions did not confer any additional benefit compared to standard care (e.g., sedation, analgesia and opioids), in relieving procedural pain during TAVI (Bruno et al. 2020), post‐TAVI (Lind et al. 2023), post‐electrophysiology and cardiac pacing procedures (Coulibaly et al. 2022) or post‐cardiac surgical pain (Rousseaux et al. 2022). This unique finding has several potential explanations. One study in this review evaluated peri‐procedural pain levels one‐day post‐procedure (Bruno et al. 2020). Ideally, pain levels should be measured ‘real time’ rather than retrospectively for a reliable result. Another potential explanation is that analgesics or opioids given during, or after the procedure/surgery, relieved the pain, or alternatively the cardiac procedures/surgery may not have been particularly painful. This explanation is supported by the fact that pain levels were low to moderate in both the VR and control groups in the studies in our meta‐analysis (Bruno et al. 2020; Coulibaly et al. 2022; Lind et al. 2023; Rousseaux et al. 2022). VR may be more effective for pain management during acutely painful procedures (Chan et al. 2018). Further research is needed to understand which cardiac procedures may be most amenable to the use of VR and at what stage in the patient journey.

Another explanation for our results could be that the VR headset was removed by 30%–62% of participants due to discomfort (Bruno et al. 2020; Coulibaly et al. 2022). The reduced duration of the intervention would therefore limit its effect. In addition, there is some evidence that VR interventions for reducing acute or chronic pain may be more potent in paediatric populations than adults (Huang et al. 2022). To be effective, VR interventions for adults may require more intense immersive content to increase levels of distraction leading to a reduction in perceived pain. Understanding why VR may potentially be more effective in children than adults warrant further investigation.

### 4.4 VR Interventions and Other Patient Outcomes

Our narrative synthesis aimed to identify the potential benefits of VR interventions on other patient outcomes linked to domains in the COMET typology (Dodd et al. 2018). Findings suggest that VR interventions have positive effects on outcomes related to cognitive functioning (procedural knowledge) and physical functioning (physical performance). Inconsistent results were found for outcomes related to emotional functioning and wellbeing (psychological distress, energy, fatigue, tense/calm), delivery of care (physical comfort, discomfort, perceived duration of procedure, satisfaction with procedure, and VR tolerance) and physiological outcomes (pulmonary function, haemodynamic parameters). The multiple types of VR interventions and the wide variety of outcomes used to measure outcomes within each COMET domain may explain inconsistent results.

Our findings suggest that VR‐facilitated education offers a more effective way to educate patients about their procedure than paper‐based written materials. This is important because more knowledgeable cardiac patients tend to have lower levels of pre‐procedural anxiety and are more satisfied with their care (Delewi et al. 2017; Hibbard and Greene 2013). Ensuring patients are fully informed about a procedure is also an ethical and legal requirement of informed consent (General Medical Council 2008) and essential for high‐quality shared decision‐making (Sepucha et al. 2013). However, many patients often misunderstand key information (e.g., potential outcomes) about cardiac procedures (Astin et al. 2020), which might partly be explained by inadequate procedural information. Paper‐based information about heart disease and procedures are often not written for people with low levels of health literacy (Zowalla, Pobiruchin, and Wiesner 2018). This is problematic because 47% of people in Europe have insufficient or problematic health literacy levels (Sorensen et al. 2015). The effectiveness of VR‐facilitated education on cardiac procedural/surgical knowledge in people with low health literacy levels warrants further research. However, VR‐facilitated education requires high capital costs and is potentially more time‐consuming per patient consultation (van der Kruk et al. 2022). The cost‐effectiveness of VR‐facilitated education also warrants further research.

Findings from our synthesis indicate that inpatient cardiac rehabilitation augmented with VR‐based exercise is more effective than usual care post‐CABG and surgical valve replacement, for improving physical functioning. VR‐based exercise rehabilitation also improved pulmonary function (Lima et al. 2020) and heart rate variability (Ribeiro et al. 2021). This is important because greater recovery of patients' daily functioning before discharge home after cardiac surgery reduces hospital readmission and 5‐year all‐cause mortality rates (Marcassa, Giordano, and Giannuzzi 2016).

Our findings consolidate previous reviews about the benefits of outpatient VR‐augmented rehabilitation (Bouraghi et al. 2023; Chen et al. 2022). Evidence shows that multidisciplinary inpatient cardiac rehabilitation incorporating education, exercise and counselling is more effective for physical and psychological recovery post‐cardiac surgery than exercise‐based in‐patient cardiac rehabilitation alone (Ogawa et al. 2021).

Future research should therefore evaluate whether the addition of other types of VR interventions (i.e., educational, relaxational and distraction) into inpatient cardiac rehabilitation exerts additional benefit than VR‐based exercise rehabilitation and conventional physical therapy alone.

### 4.5 How VR Interventions Work

The VR interventions used across the studies appeared to draw upon different mechanisms to exert an effect; through education, relaxation, distraction or by promoting rehabilitation. VR‐facilitated education provided key information about the day of the procedure. Receiving information about a procedure tends to reduce patient anxiety levels because uncertainties or misunderstandings are addressed (Stucky and Vortman 2022). In agreement, RCTs included in our meta‐analysis found that higher procedural knowledge scores were significantly associated with lower pre‐procedural anxiety (Grab et al. 2023). Two RCTs in our meta‐analysis reported significantly reduced pre‐procedural anxiety levels together with greater procedural knowledge following VR‐facilitated education, compared with usual care (paper‐based information) (Chang et al. 2021; Grab et al. 2023). This suggests that VR‐facilitated patient education reduces pre‐procedural anxiety more effectively than paper‐based information, potentially because people are more engaged during an immersive and interactive learning experience leading to better knowledge retention (Wu, Yu, and Gu 2020).

In our review, VR‐based relaxation and distraction interventions reduced patient anxiety, and VR‐based exercise rehabilitation improved physical functioning. VR‐based relaxation incorporates techniques, such as guided imagery and music, which have been shown to reduce anxiety by triggering parasympathetic nervous activity and changing thought patterns (Wang et al. 2022). VR‐based distraction alters brain activity (increases cognitive load) as patients' attention is diverted away from the anxiety‐provoking environment during a procedure (Redlinger and Shao 2021). VR‐augmented cardiac rehabilitation is more enjoyable so adherence is higher, leading to greater patient benefits (Chen et al. 2022).

Overall, our findings highlighted a gap in the research evidence about which VR intervention (type, content, dosage) confers the most benefit to patients. VR interventions may need to be tailored to target a specific outcome (anxiety, knowledge, physical function).

Just over one third of studies included in our review did not provide technical information about the VR headset and it was not always apparent whether concurrent audio was included. Incorporating both audio and visual in VR increases the sense of immersion and may therefore be more beneficial (Zhang and Song 2022).

To progress our understanding, it is important that the components of the VR interventions are described in sufficient detail. We recommend that the Tidier checklist (Hoffmann et al. 2014) is implemented to achieve this in future studies.

### 4.6 Strengths and Limitations of Review

A strength of our review is the comprehensive search of the international literature across five databases with no restrictions, which increases the breadth of studies included in the review. Multiple reviewers were involved in each stage of the review process, which reduces selection and reporting bias. The inclusion of sensitivity analysis alongside the primary meta‐analyses facilitated confirmation that no single study was excessively influencing findings.

A limitation of the review is that all studies included in the meta‐analyses were rated as having ‘some’ or ‘high’ risk of bias and sample size was low in some studies. Similarly, most studies included in the narrative synthesis were rated as ‘poor quality’ or having ‘some concerns’. Studies evaluating applied health interventions inherently don't score very well because blinded allocation is not possible and intervention effectiveness is often measured using patient‐reported outcomes, so blinded outcome measurement is not possible. Future RCTs should consult the RoB2 tool (Sterne et al. 2019) during the study design phase to improve bias ratings. Publishing a full protocol/analysis plan will facilitate a higher bias rating in domain five of the tool (bias in selection of the reported result). To improve the bias and quality of studies, future RCTs should ensure the randomisation allocation sequence is concealed and quasi‐experimental studies should include control groups and have an adequate sample size calculated using power analysis, to provide confidence in the findings. Despite the limitations this review provides a robust overview about the potential benefit of VR in cardiology settings that can inform policy, practice, and education.

## 5 Conclusion

Patients undergoing elective cardiac surgical and interventional procedures may benefit from VR interventions via reduced anxiety before and during procedures and increased pre‐procedural knowledge and postoperative physical function. VR interventions may also improve emotional functioning and wellbeing, delivery of care and physiological outcomes, but the evidence is inconsistent. VR interventions, as an adjunct to analgesia, did not enhance pain relief in the studies in our meta‐analysis.

Future research is needed to explore the type, content and dose of VR interventions that are most effective for improving outcomes in cardiac patients. VR was the only XR technology to be evaluated in patients undergoing cardiac procedures. Therefore, the application of augmented or mixed reality interventions in this setting should be explored.

## 6 Relevance to Clinical Practice

Our review findings indicate that the cardiac nurses' role can be supported by VR interventions to enhance levels of psychosocial support and education for patients undergoing elective cardiac surgical and interventional procedures. These care activities are among the first to be ‘rationed’ when resources are insufficient to meet the demands of the role (Wagner‐Łosieczka et al. 2023). Finding ways of integrating effective digital technologies may offset unmet care needs caused by workforce shortages and increased demand for cardiac treatments (iData Research 2021; World Health Organisation 2020).

## Author Contributions

Conceptualisation: E.H., F.A., S.F. and H.L. Methodology: E.H., F.A., S.F. and H.L. Investigation: E.H., F.A., F.E., H.L., J.S., S.F. and S.G. Visualisation: E.H. and J.S. Project administration: E.H. Writing – original draft: E.H., F.A., H.L., J.S. and S.F. Writing – review and editing: E.H., F.A., F.E., H.L., J.S. and S.F.

## Conflicts of Interest

The authors declare no conflicts of interest.

## Supporting information


Data S1.

Data S2.

## Data Availability

The data that support the findings of this study are available from the corresponding author upon reasonable request.
