# Supplementary material for: Do Extended Reality Interventions Benefit Patients Undergoing Elective Cardiac Surgical and Interventional Procedures? A Systematic Review and Meta‐analysis
Source: J Clin Nurs. 2024 Dec 12;34(4):1465–92. doi: 10.1111/jocn.17578 (PMC11933515; doi:10.1111/jocn.17578)
Supplement: Supplementary file 2 — Data S2. [file JOCN-34-1465-s002.docx]

**Supplementary File**

**Do extended reality interventions benefit patients undergoing elective cardiac surgical and interventional procedures? A systematic review and meta-analysis.**

**Contents**

[1. Table S1: PRISMA 2020 Checklist^1^ 2](#_Toc158221531)

[2. Search terms 6](#_Toc158221532)

[MEDLINE (EBSCO) 6](#_Toc158221533)

[CINAHL (EBSCO) 7](#_Toc158221534)

[PsycInfo (ProQuest) 8](#_Toc158221535)

[Cochrane Central Register of Controlled Trials (CENTRAL, The Cochrane Library) 8](#_Toc158221536)

[Scopus 9](#_Toc158221537)

[3. Table S2: Modified NHLBI Quality Assessment for Non-randomised Studies. 11](#_Toc158221538)

[4. References 13](#_Toc158221539)

# Table S1: PRISMA 2020 Checklist^1^

| **Section and Topic** | **Item #** | **Checklist item** | **Location where item is reported** |
| --- | --- | --- | --- |
| **TITLE** | | |  |
| Title | 1 | Identify the report as a systematic review. | Title page |
| **ABSTRACT** | | |  |
| Background-Objectives | 2a | Provide an explicit statement of the main objective(s) or question(s) the review addresses. | Aim |
| Methods- Eligibility criteria | 2b | Specify the inclusion and exclusion criteria for the review. | Limited by word count and journal criteria for structure |
| Methods- Information sources | 2c | Specify the information sources (e.g. databases, registers) used to identify studies and the date when each was last searched. | Data sources |
| Methods- Risk of bias | 2d | Specify the methods used to assess risk of bias in the included studies. | Methods |
| Methods- Synthesis of results | 2e | Specify the methods used to present and synthesise results. | Methods |
| Results- Included studies | 2f | Give the total number of included studies and participants and summarise relevant characteristics of studies. | Results |
| Results- Synthesis of results | 2g | Present results for main outcomes, preferably indicating the number of included studies and participants for each. If meta-analysis was done, report the summary estimate and confidence/credible interval. If comparing groups, indicate the direction of the effect (i.e. which group is favoured). | Results |
| Discussion- Limitations of evidence | 2h | Provide a brief summary of the limitations of the evidence included in the review (e.g. study risk of bias, inconsistency and imprecision). | Risk of bias stated in Results |
| Discussion-Interpretation | 2i | Provide a general interpretation of the results and important implications. | Conclusions and relevance to clinical practice |
| Other- Funding | 2j | Specify the primary source of funding for the review. | n/a |
| Other- Registration | 2k | Provide the register name and registration number. | n/a |
| **INTRODUCTION** | | |  |
| Rationale | 3 | Describe the rationale for the review in the context of existing knowledge. | Section 1. Introduction |
| Objectives | 4 | Provide an explicit statement of the objective(s) or question(s) the review addresses. | Section 1. Introduction |
| **METHODS** | | |  |
| Eligibility criteria | 5 | Specify the inclusion and exclusion criteria for the review and how studies were grouped for the syntheses. | Section 2.3. Eligibility criteria and table 1. |
| Information sources | 6 | Specify all databases, registers, websites, organisations, reference lists and other sources searched or consulted to identify studies. Specify the date when each source was last searched or consulted. | Section 2.2. Search strategy |
| Search strategy | 7 | Present the full search strategies for all databases, registers and websites, including any filters and limits used. | Section 2.2. Search strategy and supplementary file |
| Selection process | 8 | Specify the methods used to decide whether a study met the inclusion criteria of the review, including how many reviewers screened each record and each report retrieved, whether they worked independently, and if applicable, details of automation tools used in the process. | Section 2.4. Study selection |
| Data collection process | 9 | Specify the methods used to collect data from reports, including how many reviewers collected data from each report, whether they worked independently, any processes for obtaining or confirming data from study investigators, and if applicable, details of automation tools used in the process. | Section 2.5. Data extraction |
| Data items | 10a | List and define all outcomes for which data were sought. Specify whether all results that were compatible with each outcome domain in each study were sought (e.g. for all measures, time points, analyses), and if not, the methods used to decide which results to collect. | Section 2.5. Data extraction and table 1. |
|  | 10b | List and define all other variables for which data were sought (e.g. participant and intervention characteristics, funding sources). Describe any assumptions made about any missing or unclear information. | Section 2.5. Data extraction |
| Study risk of bias assessment | 11 | Specify the methods used to assess risk of bias in the included studies, including details of the tool(s) used, how many reviewers assessed each study and whether they worked independently, and if applicable, details of automation tools used in the process. | Section 2.6. Quality assessment |
| Effect measures | 12 | Specify for each outcome the effect measure(s) (e.g. risk ratio, mean difference) used in the synthesis or presentation of results. | Section 2.7. Data analysis and synthesis |
| Synthesis methods | 13a | Describe the processes used to decide which studies were eligible for each synthesis (e.g. tabulating the study intervention characteristics and comparing against the planned groups for each synthesis (item #5)). | Section 2.7. Data analysis and synthesis |
|  | 13b | Describe any methods required to prepare the data for presentation or synthesis, such as handling of missing summary statistics, or data conversions. | Section 2.7. Data analysis and synthesis |
|  | 13c | Describe any methods used to tabulate or visually display results of individual studies and syntheses. | Section 2.7. Data analysis and synthesis |
|  | 13d | Describe any methods used to synthesize results and provide a rationale for the choice(s). If meta-analysis was performed, describe the model(s), method(s) to identify the presence and extent of statistical heterogeneity, and software package(s) used. | Section 2.7. Data analysis and synthesis |
|  | 13e | Describe any methods used to explore possible causes of heterogeneity among study results (e.g. subgroup analysis, meta-regression). | Section 2.7. Data analysis and synthesis |
|  | 13f | Describe any sensitivity analyses conducted to assess robustness of the synthesized results. | Section 2.7. Data analysis and synthesis |
| Reporting bias assessment | 14 | Describe any methods used to assess risk of bias due to missing results in a synthesis (arising from reporting biases). | Section 2.7. Data analysis and synthesis |
| Certainty assessment | 15 | Describe any methods used to assess certainty (or confidence) in the body of evidence for an outcome. | n/a |
| **RESULTS** | | |  |
| Study selection | 16a | Describe the results of the search and selection process, from the number of records identified in the search to the number of studies included in the review, ideally using a flow diagram. | Section 3.1 Study selection and Figure 1 |
|  | 16b | Cite studies that might appear to meet the inclusion criteria, but which were excluded, and explain why they were excluded. | n/a |
| Study characteristics | 17 | Cite each included study and present its characteristics. | Table 2 and Sections 3.2. Overview of Studies and 3.3. Characteristics of VR interventions |
| Risk of bias in studies | 18 | Present assessments of risk of bias for each included study. | Section 3.4 Quality of studies and Figure 2 |
| Results of individual studies | 19 | For all outcomes, present, for each study: (a) summary statistics for each group (where appropriate) and (b) an effect estimate and its precision (e.g. confidence/credible interval), ideally using structured tables or plots. | Tables 2 |
| Results of syntheses | 20a | For each synthesis, briefly summarise the characteristics and risk of bias among contributing studies. | Section 3.4 Quality of studies, 3.5.1, 3.6.1, 3.7.1 |
|  | 20b | Present results of all statistical syntheses conducted. If meta-analysis was done, present for each the summary estimate and its precision (e.g. confidence/credible interval) and measures of statistical heterogeneity. If comparing groups, describe the direction of the effect. | Sections 3.5 – 3.7 |
|  | 20c | Present results of all investigations of possible causes of heterogeneity among study results. | Sections 3.5 – 3.7 |
|  | 20d | Present results of all sensitivity analyses conducted to assess the robustness of the synthesized results. | Sections 3.5 – 3.7 |
| Reporting biases | 21 | Present assessments of risk of bias due to missing results (arising from reporting biases) for each synthesis assessed. | n/a |
| Certainty of evidence | 22 | Present assessments of certainty (or confidence) in the body of evidence for each outcome assessed. | n/a |
| **DISCUSSION** | | |  |
| Discussion | 23a | Provide a general interpretation of the results in the context of other evidence. | Section 4.0. Discussion |
|  | 23b | Discuss any limitations of the evidence included in the review. | Section 4.1. Strengths and limitations of review |
|  | 23c | Discuss any limitations of the review processes used. | Section 4.1. Strengths and limitations of review |
|  | 23d | Discuss implications of the results for practice, policy, and future research. | Sections 5-6. Conclusion and Relevance to clinical practice. |
| **OTHER INFORMATION** | | |  |
| Registration and protocol | 24a | Provide registration information for the review, including register name and registration number, or state that the review was not registered. | Section 2.1. Design. |
|  | 24b | Indicate where the review protocol can be accessed, or state that a protocol was not prepared. | Section 2.1. Design. |
|  | 24c | Describe and explain any amendments to information provided at registration or in the protocol. | Change of author affiliation and update of the quality appraisal tools used. |
| Support | 25 | Describe sources of financial or non-financial support for the review, and the role of the funders or sponsors in the review. | Acknowledgements and funding information |
| Competing interests | 26 | Declare any competing interests of review authors. | Conflicts of Interest |
| Availability of data, code and other materials | 27 | Report which of the following are publicly available and where they can be found: template data collection forms; data extracted from included studies; data used for all analyses; analytic code; any other materials used in the review. | Data availability statement |

# Search terms

## MEDLINE (EBSCO)

**S1** (MM "Virtual Reality Exposure Therapy") OR (MM "Virtual Reality+") OR (MM "Augmented Reality")

**S2** AB ( "audio" OR "immersive sound" OR "immersive experience" OR "virtual reality" OR "augmented reality" OR "mixed reality" OR “virtual world” OR "virtual therapy" OR "virtual environment" OR "virtual auditory" OR "audio visual" OR “extended reality” OR “head mounted”) OR TI ( "audio" OR "immersive sound" OR "immersive experience" OR "virtual reality" OR "augmented reality" OR "mixed reality" OR “virtual world” OR "virtual therapy" OR "virtual environment" OR "virtual auditory" OR "audio visual" OR “extended reality” OR “head mounted”)

**S3** (MM "Catheter Ablation+") OR (MM "Cardiac Catheterization+") OR (MM "Angioplasty+") OR (MM "Angioplasty, Balloon+") OR (MM "Angioplasty, Laser+") OR (MM "Percutaneous Coronary Intervention+") OR (MM "Myocardial Revascularization+") OR (MM "Cardiac Surgical Procedures+") OR (MM "Cardiac Valve Annuloplasty+") OR (MM "Heart Bypass, Right+") OR (MM "Heart Valve Prosthesis Implantation+") OR (MM "Transcatheter Aortic Valve Replacement") OR (MM "Coronary Artery Bypass+") OR (MM "Pacemaker, Artificial+") OR (MM "Cardiac Resynchronization Therapy Devices") OR (MM "Defibrillators+") OR (MM "Myocardial Reperfusion") OR (MM "Cardiovascular Surgical Procedures+") OR (MM "Diagnostic Techniques, Cardiovascular+")

**S4** TI ( (cardiac OR heart OR cardiology OR coronary OR cardiovascular OR myocardial) N3 (procedure* OR surgery OR imaging OR intervention* OR catheteri?ation OR revasculari?ation OR reperfusion OR bypass OR device* OR resynchroni?ation) ) OR AB ( (cardiac OR heart OR cardiology OR coronary OR cardiovascular OR myocardial) N3 (procedure* OR surgery OR imaging OR intervention* OR catheteri?ation OR revasculari?ation OR reperfusion OR bypass OR device* OR resynchroni?ation) )

**S5** AB ( PCI OR “percutaneous coronary intervention” OR angiogra* OR angioplasty ) OR TI ( PCI OR “percutaneous coronary intervention” OR angiogra* OR angioplasty )

**S6** AB ( ("aortic valve" OR "heart valve" OR “mitral valve”) N2 (replace* OR implant* OR repair) ) OR TI ( ("aortic valve" OR "heart valve" OR “mitral valve”) N2 (replace* OR implant* OR repair) )

**S7** AB ( pacemaker OR "implantable Cardioverter Defibrillator" OR ICD OR LVAD OR "left ventricular assist device" ) OR TI ( pacemaker OR "implantable Cardioverter Defibrillator" OR ICD OR LVAD OR "left ventricular assist device" )

**S8** AB ( "valve annuloplasty" OR valvuloplasty OR "catheter ablation" OR "coronary sinus reducer" OR "septal defect closure" OR cardioversion OR "implantable loop recorder" OR "appendage occlusion device*" OR "alcohol septal ablation" ) OR TI ( "valve annuloplasty" OR valvuloplasty OR "catheter ablation" OR "coronary sinus reducer" OR "septal defect closure" OR cardioversion OR "implantable loop recorder" OR "appendage occlusion device*" OR "alcohol septal ablation" )

**S9** S1 OR S2

**S10** S3 OR S4 OR S5 OR S6 OR S7 OR S8

**S11** S9 AND S10

## CINAHL (EBSCO)

**S1** (MM "Virtual Reality Exposure Therapy") OR (MM "Virtual Reality+") OR (MM "Augmented Reality")

**S2** AB ( "audio" OR "immersive sound" OR "immersive experience" OR "virtual reality" OR "augmented reality" OR "mixed reality" OR “virtual world” OR "virtual therapy" OR "virtual environment" OR "virtual auditory" OR "audio visual" OR “extended reality” OR “head mounted”) OR TI ( "audio" OR "immersive sound" OR "immersive experience" OR "virtual reality" OR "augmented reality" OR "mixed reality" OR “virtual world” OR "virtual therapy" OR "virtual environment" OR "virtual auditory" OR "audio visual" OR “extended reality” OR “head mounted”)

**S3** (MM "Catheter Ablation") OR (MM "Heart Catheterization+") OR (MM "Angioplasty+") OR (MM "Angioplasty, Balloon+") OR (MM "Angioplasty, Laser+") OR (MM "Percutaneous Coronary Intervention+") OR (MM "Myocardial Revascularization+") OR (MM "Heart surgery") OR (MM "Cardiac Valve Annuloplasty+") OR (MM "Heart Valve Prosthesis+") OR (MM "Transcatheter Aortic Valve Implantation") OR (MM "Coronary Artery Bypass+") OR (MM "Pacemaker, Artificial") OR (MM "Cardiac Resynchronization Therapy") OR (MM “Cardiac Pacing, Artificial+”) OR (MM "Defibrillators, Implantable") OR (MM "Myocardial Reperfusion") OR (MM "Surgery, Cardiovascular") OR (MM "Diagnosis, Cardiovascular+")

**S4** TI ( (cardiac OR heart OR cardiology OR coronary OR cardiovascular OR myocardial) N3 (procedure* OR surgery OR imaging OR intervention* OR catheteri?ation OR revasculari?ation OR reperfusion OR bypass OR device* OR resynchroni?ation) ) OR AB ( (cardiac OR heart OR cardiology OR coronary OR cardiovascular OR myocardial) N3 (procedure* OR surgery OR imaging OR intervention* OR catheteri?ation OR revasculari?ation OR reperfusion OR bypass OR device* OR resynchroni?ation) )

**S5** AB ( PCI OR “percutaneous coronary intervention” OR angiogra* OR angioplasty ) OR TI ( PCI OR “percutaneous coronary intervention” OR angiogra* OR angioplasty )

**S6** AB ( ("aortic valve" OR "heart valve" OR “mitral valve”) N2 (replace* OR implant* OR repair) ) OR TI ( ("aortic valve" OR "heart valve" OR “mitral valve”) N2 (replace* OR implant* OR repair) )

**S7** AB ( pacemaker OR "implantable Cardioverter Defibrillator" OR ICD OR LVAD OR "left ventricular assist device" ) OR TI ( pacemaker OR "implantable Cardioverter Defibrillator" OR ICD OR LVAD OR "left ventricular assist device" )

**S8** AB ( "valve annuloplasty" OR valvuloplasty OR "catheter ablation" OR "coronary sinus reducer" OR "septal defect closure" OR cardioversion OR "implantable loop recorder" OR "appendage occlusion device*" OR "alcohol septal ablation" ) OR TI ( "valve annuloplasty" OR valvuloplasty OR "catheter ablation" OR "coronary sinus reducer" OR "septal defect closure" OR cardioversion OR "implantable loop recorder" OR "appendage occlusion device*" OR "alcohol septal ablation" )

**S9** S1 OR S2

**S10** S3 OR S4 OR S5 OR S6 OR S7 OR S8

**S11** S9 AND S10

## PsycInfo (ProQuest)

**S1** MJMAINSUBJECT.EXACT("Virtual Reality Exposure Therapy") OR MJMAINSUBJECT.EXACT.EXPLODE("Virtual Reality") OR MJMAINSUBJECT.EXACT("Augmented Reality")

**S2** AB,TI( "audio" OR "immersive sound" OR "immersive experience" OR "virtual reality" OR "augmented reality" OR "mixed reality" OR “virtual world” OR "virtual therapy" OR "virtual environment" OR "virtual auditory" OR "audio visual" OR “extended reality” OR “head mounted”)

**S3** MJMAINSUBJECT.EXACT("Catheterization") OR MJMAINSUBJECT.EXACT("Heart Surgery") OR MJMAINSUBJECT.EXACT("Artificial Pacemakers") OR MJMAINSUBJECT.EXACT("Angiography") OR MJMAINSUBJECT.EXACT("Heart Valves")

**S4** AB,TI(cardiac OR heart OR cardiology OR coronary OR cardiovascular OR myocardial) Near/3 AB,TI(procedure* OR surgery OR imaging OR intervention* OR catheteri?ation OR revasculari?ation OR reperfusion OR bypass OR device* OR resynchroni?ation)

**S5** AB,TI( PCI OR “percutaneous coronary intervention” OR angiogra* OR angioplasty )

**S6** AB,TI("aortic valve" OR "heart valve" OR “mitral valve”) NEAR/2 AB,TI(replace* OR implant* OR repair)

**S7** AB,TI( pacemaker OR "implantable Cardioverter Defibrillator" OR ICD OR LVAD OR "left ventricular assist device")

**S8** AB,TI( "valve annuloplasty" OR valvuloplasty OR "catheter ablation" OR "coronary sinus reducer" OR "septal defect closure" OR cardioversion OR "implantable loop recorder" OR "appendage occlusion device*" OR "alcohol septal ablation" )

**S9** S1 OR S2

**S10** S3 OR S4 OR S5 OR S6 OR S7 OR S8

**S11** S9 AND S10

## Cochrane Central Register of Controlled Trials (CENTRAL, The Cochrane Library)

**#1** MeSH descriptor: [Virtual Reality] explode all trees

**#2** MeSH descriptor: [Virtual Reality Exposure Therapy] explode all trees

**#3** MeSH descriptor: [Augmented Reality] explode all trees

**#4** #1 OR #2 OR #3

**#5** ("audio" OR "immersive sound" OR "immersive experience" OR "virtual reality" OR "augmented reality" OR "mixed reality" OR “virtual world” OR "virtual therapy" OR "virtual environment" OR "virtual auditory" OR "audio visual" OR “extended reality” OR “head mounted”):ti,ab,kw (Word variations have been searched)

**#6** MeSH descriptor: [Catheter Ablation] explode all trees

**#7** MeSH descriptor: [Cardiac Catheterization] explode all trees

**#8** MeSH descriptor: [Angioplasty] explode all trees

**#9** MeSH descriptor: [Angioplasty, Balloon, Coronary] explode all trees

**#10** MeSH descriptor: [Angioplasty, Balloon, Laser-Assisted] explode all trees

**#11** MeSH descriptor: [Percutaneous Coronary Intervention] explode all trees

**#12** MeSH descriptor: [Myocardial Revascularization] explode all trees

**#13** MeSH descriptor: [Cardiac Surgical Procedures] explode all trees

**#14** MeSH descriptor: [Cardiac Valve Annuloplasty] explode all trees

**#15** MeSH descriptor: [Heart Bypass, Right] explode all trees

**#16** MeSH descriptor: [Heart Valve Prosthesis Implantation] explode all trees

**#17** MeSH descriptor: [Transcatheter Aortic Valve Replacement] explode all trees

**#18** MeSH descriptor: [Coronary Artery Bypass] explode all trees

**#19** MeSH descriptor: [Pacemaker, Artificial] explode all trees

**#20** MeSH descriptor: [Cardiac Resynchronization Therapy Devices] explode all trees

**#21** MeSH descriptor: [Defibrillators] explode all trees

**#22** MeSH descriptor: [Myocardial Reperfusion] explode all trees

**#23** MeSH descriptor: [Cardiovascular Surgical Procedures] explode all trees

**#24** MeSH descriptor: [Diagnostic Techniques, Cardiovascular] explode all trees

**#25** #6 OR #7 OR #8 OR #9 OR #10 OR #11 OR #12 OR #13 OR #14 OR #15 OR #16 OR #17 OR #18 OR #19 OR #20 OR #21 OR #22 OR #23 OR #24

**#26** ((cardiac OR heart OR cardiology OR coronary OR cardiovascular OR myocardial) near/3 (procedure* OR surgery OR imaging OR intervention* OR catheteri?ation OR revasculari?ation OR reperfusion OR bypass OR device* OR resynchroni?ation)):ti,ab,kw (Word variations have been searched)

**#27** (PCI OR “percutaneous coronary intervention” OR angiogra* OR angioplasty):ti,ab,kw (Word variations have been searched)

**#28** (("aortic valve" OR "heart valve" OR “mitral valve”) near/2 (replace* OR implant* OR repair)):ti,ab,kw (Word variations have been searched)

**#29** (pacemaker OR "implantable Cardioverter Defibrillator" OR ICD OR LVAD OR "left ventricular assist device"):ti,ab,kw (Word variations have been searched)

**#30** ("valve annuloplasty" OR valvuloplasty OR "catheter ablation" OR "coronary sinus reducer" OR "septal defect closure" OR cardioversion OR "implantable loop recorder" OR "appendage occlusion device*" OR "alcohol septal ablation"):ti,ab,kw (Word variations have been searched)

**#31** #4 OR #5

**#32** #25 OR #26 OR #27 OR #28 OR #29 OR #30

**#33** #31 AND #32

## Scopus

**S1** INDEXTERMS("virtual reality" OR "Virtual Reality Exposure Therapy" OR "augmented reality" OR "extended reality" OR "mixed reality")

**S2** TITLE-ABS("audio" OR "immersive sound" OR "immersive experience" OR "virtual reality" OR "augmented reality" OR "mixed reality" OR “virtual world” OR "virtual therapy" OR "virtual environment" OR "virtual auditory" OR "audio visual" OR “extended reality” OR “head mounted”)

**S3** INDEXTERMS("Catheter Ablation" OR "Cardiac Catheterization” OR "Angioplasty" OR "Angioplasty, Balloon" OR "Angioplasty, Laser" OR "Percutaneous Coronary Intervention" OR "Myocardial Revascularization" OR "Cardiac Surgical Procedures" OR "Cardiac Valve Annuloplasty" OR "Heart Bypass, Right" OR “Heart Valve Prosthesis Implantation" OR "Transcatheter Aortic Valve Replacement" OR "Coronary Artery Bypass" OR "Pacemaker, Artificial" OR "Cardiac Resynchronization Therapy Devices" OR "Defibrillators" OR "Myocardial Reperfusion" OR "Cardiovascular Surgical Procedures” OR "Diagnostic Techniques, Cardiovascular")

**S4** TITLE-ABS((cardiac W/3 procedure*) OR (cardiac W/3 surgery) OR (cardiac W/3 imaging) OR (cardiac W/3 intervention*) OR (cardiac W/3 catheteri?ation) OR (cardiac W/3 revasculari?ation) OR (cardiac W/3 reperfusion) OR (cardiac W/3 bypass) OR (cardiac W/3 device*) OR (cardiac W/3 resynchroni?ation))

**S5** TITLE-ABS((heart W/3 procedure*) OR (heart W/3 surgery) OR (heart W/3 imaging) OR (heart W/3 intervention*) OR (heart W/3 catheteri?ation) OR (heart W/3 revasculari?ation) OR (heart W/3 reperfusion) OR (heart W/3 bypass) OR (heart W/3 device*) OR (heart W/3 resynchroni?ation))

**S6** TITLE-ABS((cardiology W/3 procedure*) OR (cardiology W/3 surgery) OR (cardiology W/3 imaging) OR (cardiology W/3 intervention*) OR (cardiology W/3 catheteri?ation) OR (cardiology W/3 revasculari?ation) OR (cardiology W/3 reperfusion) OR (cardiology W/3 bypass) OR (cardiology W/3 device*) OR (cardiology W/3 resynchroni?ation))

**S7** TITLE-ABS((coronary W/3 procedure*) OR (coronary W/3 surgery) OR (coronary W/3 imaging) OR (coronary W/3 intervention*) OR (coronary W/3 catheteri?ation) OR (coronary W/3 revasculari?ation) OR (coronary W/3 reperfusion) OR (coronary W/3 bypass) OR (coronary W/3 device*) OR (coronary W/3 resynchroni?ation))

**S8** TITLE-ABS((cardiovascular W/3 procedure*) OR (cardiovascular W/3 surgery) OR (cardiovascular W/3 imaging) OR (cardiovascular W/3 intervention*) OR (cardiovascular W/3 catheteri?ation) OR (cardiovascular W/3 revasculari?ation) OR (cardiovascular W/3 reperfusion) OR (cardiovascular W/3 bypass) OR (cardiovascular W/3 device*) OR (cardiovascular W/3 resynchroni?ation))

**S9** TITLE-ABS((myocardial W/3 procedure*) OR (myocardial W/3 surgery) OR (myocardial W/3 imaging) OR (myocardial W/3 intervention*) OR (myocardial W/3 catheteri?ation) OR (myocardial W/3 revasculari?ation) OR (myocardial W/3 reperfusion) OR (myocardial W/3 bypass) OR (myocardial W/3 device*) OR (myocardial W/3 resynchroni?ation))

**S10** TITLE-ABS(PCI OR “percutaneous coronary intervention” OR angiogra* OR angioplasty)

**S11** TITLE-ABS("aortic valve" W/2 replace*) OR (“aortic valve” W/2 implant*) OR (“aortic valve” W/2 repair)

**S12** TITLE-ABS("heart valve" W/2 replace*) OR (“heart valve” W/2 implant*) OR (“heart valve” W/2 repair)

**S13** TITLE-ABS("mitral valve" W/2 replace*) OR (“mitral valve” W/2 implant*) OR (“mitral valve” W/2 repair)

**S14** TITLE-ABS(pacemaker OR "implantable Cardioverter Defibrillator" OR ICD OR LVAD OR "left ventricular assist device")

**S15** TITLE-ABS("valve annuloplasty" OR valvuloplasty OR "catheter ablation" OR "coronary sinus reducer" OR "septal defect closure" OR cardioversion OR "implantable loop recorder" OR "appendage occlusion device*" OR "alcohol septal ablation")

**S16** S1 OR S2

**S17** S3 OR S4 OR S5 OR S6 OR S7 OR S8 OR S9 OR S10 OR S11 OR S12 OR S13 OR S14 OR S15

**S18** S16 AND S17

# Table S2: Modified NHLBI Quality Assessment for Non-randomised Studies.

**Name of assessor:**

**Study being assessed (first author and study title):**

| **Criteria** | **Yes (1 point)** | **No (0 point)** | **Partially (0.5 points) or not applicable (N/A) (0 points)** | |
| --- | --- | --- | --- | --- |
| 1. Was the study question or objective clearly stated?   *Notes: Answer yes or no* |  |  |  | |
| 1. Were eligibility/selection criteria for the study population prespecified and clearly described?   *Notes:* *whom to recruit, from where, and from what time-period. For yes, need to state all 3 criteria. Answer no if no information is given. Answer partially if some information is provided.* |  |  |  | |
| 1. Were the participants in the study representative of those who would be eligible for the test/service/intervention in the general or clinical population of interest?   *Notes: The participants in the study should be generally representative of the population in which the intervention will be broadly applied. Studies on small demographic subgroups may raise concerns about how the intervention will affect broader populations of interest. If the sample is a specific population (e.g. men only) then answer no as this would not be representative of everyone who would have a cardiac procedure and could receive VR intervention.* |  |  |  | |
| 1. Was the sample size sufficiently large to provide confidence in the findings?   *Notes: Answer yes if a target sample size was stated using power analysis and this target was achieved.* |  |  |  | |
| 1. Was there a control group of participants that did not receive the VR intervention?   *Notes: Answer yes if a control group was included in the study design and this group received usual care. Answer partially if a usual care control group was not included but a comparison group of a different intervention (e.g., hypnosis group, medication group) was included in the study design. Answer no if neither a control nor comparison group was included in the study design.* |  |  |  | |
| 1. Were the groups similar at baseline on important characteristics that could affect outcomes (e.g., baseline anxiety higher in one group)?   *Notes: Answer n/a if the study only has one group. Answer yes if no significant differences were noted. Answer no if significant differences were noted. Answer partially if there was a noticeable difference with a trend towards significance. Answer partially if multiple baseline differences were noted for characteristics that if on their own would not affect outcomes, but when combined with other variables could have an affect (e.g., higher number of men in one group would be no but if this group were also significantly older and had more severe disease, this would be partially)* |  |  |  | |
| 1. Were the outcome measures appropriate?   *Notes: Validated scales, Likert scales, or physiological measurement tools (e.g. HR monitor) would be appropriate. Researcher generated questionnaires would not be appropriate. Answer yes if all measures were appropriate; no if none of the measures were appropriate; partially if some but not all measures were appropriate.* |  |  |  | |
| 1. Did the statistical methods examine changes in outcome measures from either before to after the intervention or at post-intervention between groups and were the values and p-values reported?   *Notes: Answer yes if changes between timepoints or groups were examined and both the outcome measure descriptives (e.g., mean, SD or Median, IQR) was reported along with the p value.*  *Answer partially if only some of the above was reported.*  *Answer no for studies that assess outcome measures at one time point only and have only one group (i.e. no control group).* |  |  |  | |
| **Quality Rating:**  **Each YES response scores 1 point; NO response scores 0 points; partially response scores 0.5 points; N/A response scores 0 points.**  **6.5-8 points= Good quality**  **4-6 points= Some Concerns**  **0-3.5 points= Poor quality** | | | |  |
| **Score:** | | | |  |
| **Overall rating:** | | | |  |
| **Additional Comments (If POOR, please state why):** | | | |  |

# References

1. Page MJ, McKenzie JE, Bossuyt PM, Boutron I, Hoffmann TC, Mulrow CD, et al. The PRISMA 2020 statement: an updated guideline for reporting systematic reviews. *BMJ (Clinical research ed)* 2021; 372: n71. DOI: <https://doi.org/10.1136/bmj.n71>.
